# Morphology and ultrastructure of pharyngeal sense organs of *Drosophila* larvae

**DOI:** 10.7554/eLife.108036

**Published:** 2026-10-23

**Authors:** Vincent Richter, Tilman Triphan, Albert Cardona, Andreas S Thum

**Affiliations:** 1 Department of Genetics, University of Leipzig, Institute for Biology Leipzig Germany; 2 MRC Laboratory of Molecular Biology, Cambridge Biomedical Campus Cambridge United Kingdom; 3 Department of Physiology, Development and Neuroscience, University of Cambridge Cambridge United Kingdom; 4 German Centre for Integrative Biodiversity Research (iDiv) Halle-Jena-Leipzig Leipzig Germany; Universidad de Buenos Aires - CONICET Argentina; Tata Institute for Genetics and Society India

**Keywords:** sensilla, peripheral nervous system, electron microscopy, chemosensation, mechanosensation, *D. melanogaster*

## Abstract

This study provides a comprehensive ultrastructural analysis of the pharyngeal sensory system in *Drosophila melanogaster* larvae, focusing on the four major pharyngeal sense organs: the ventral pharyngeal sensilla (VPS), dorsal pharyngeal sensilla (DPS), dorsal pharyngeal organ (DPO), and posterior pharyngeal sensilla (PPS). Our analysis revealed 15 sensilla across these organs, comprising four mechanosensory, nine chemosensory, and two dual-function sensilla. We identified 35 Type I neurons (6 mechanosensory and 29 chemosensory) and 6 Type II neurons with putative chemosensory functions. Additional sensory structures, including papilla sensilla and chordotonal organs in the cephalopharyngeal region, were characterized. This detailed mapping and classification of pharyngeal sensory structures completes their structural characterization and provides a foundation for future anatomical and functional studies of sensory perception in insects. This work represents a significant step toward a complete analysis of the larval sensory system, providing new opportunities for investigating how an organism processes sensory information to navigate and interact with its environment.

## Introduction

The larva of the fruit fly (*Drosophila melanogaster*) has emerged as a key model system for investigating how organisms process sensory information (reviewed in Gerber and Stocker, 2007; Melcher et al., 2007; Apostolopoulou et al., 2015; Joseph and Carlson, 2015; Rimal and Lee, 2018; Widmann et al., 2018; Thum and Gerber, 2019). Among all sensory modalities, assessing food quality and innocuousness is most critical for survival, particularly in holometabolous insects like *Drosophila*, as its larval stage represents the primary feeding phase during which the organism ingests substantial amounts of food to sustain rapid growth and prepare for metamorphosis (Amrein and Thorne, 2005). By taking advantage of light microscopy, volume electron microscopy (EM), and 3D imaging analysis, recent studies provided detailed ultrastructural characterization of the various components of the larval *Drosophila* sensory system (Grueber et al., 2002; Python and Stocker, 2002; Caldwell et al., 2003; Fishilevich et al., 2005; Kreher et al., 2005; Hwang et al., 2007; Xiang et al., 2010; Kwon et al., 2011; Apostolopoulou et al., 2014; Stewart et al., 2015; Berck et al., 2016; Larderet et al., 2017; Rist and Thum, 2017; Miroschnikow et al., 2018; Hartenstein et al., 2019; Hernandez-Nunez et al., 2021; Richter et al., 2024; Schoofs et al., 2024). These descriptions encompass the peripheral sensory system, including the four cephalic sensory organs, the terminal organ (TO), dorsal organ (DO), ventral organ (VO), and labial organ (LO), as well as the enteric nervous system, comprising the esophageal ganglion, hypercerebral ganglion, and proventricular ganglion. Additionally, they include all external body wall sensilla, proprioceptive chordotonal organs, photoreceptors, and most multidendritic sensory neurons that can be found within the larval body segments. In contrast, a detailed ultrastructural description at the level of individual sensilla and cellular resolution is lacking for the pharyngeal sensory system, despite several studies focusing mainly on the general anatomical organization and the mapping of receptor gene expression (Singh and Singh, 1984; Singh, 1997; Python and Stocker, 2002; Gendre et al., 2004; Colomb et al., 2007; Kwon et al., 2011; Apostolopoulou et al., 2016; Miroschnikow et al., 2018). This gap reflects, at least in part, the technical challenges associated with the three-dimensional visualization of deep internal structures by EM. These limitations are now increasingly overcome by advances in volume EM (White et al., 1986; Eberle et al., 2015; Xu et al., 2017; Peddie et al., 2022; Wang et al., 2021; Collinson et al., 2023; Takemura et al., 2023; Winding et al., 2023; Peale et al., 2024). Accordingly, we have conducted an ultrastructural analysis of the four principal sensory organs of the larval pharynx using volume EM. These organs include the ventral pharyngeal sensilla (VPS), dorsal pharyngeal sensilla (DPS), dorsal pharyngeal organ (DPO), and posterior pharyngeal sensilla (PPS) (Python and Stocker, 2002; Gendre et al., 2004).

In larvae, Gendre et al., 2004, comprehensively described the four pharyngeal sensory organs and their developmental fate: the DPS and the DPO develop into the adult labral and ventral cibarial sensory organs (LSO and VCSO), respectively; the posterior PPS give rise to the adult dorsal cibarial sensory organ, whereas the VPS, derived from the labial segment, undergo apoptosis during metamorphosis. These structures were characterized by detailed transmission EM and morphological analysis (Stocker and Schorderet, 1981; Naresh Singh and Nayak, 1985; Nayak and Singh, 1983; Singh, 1997; Kendroud et al., 2018). However, these earlier studies provided only EM-based schematics and a general organization of pharyngeal sensilla, without the comprehensive high-resolution volumetric analysis now achievable through modern EM methods.

Behavioral, physiological, and functional studies have shown that the larval pharyngeal system is instructing taste-guided behaviors. For instance, a single Gr93a-positive neuron pair located in the DPS instructs caffeine-dependent choice behavior and aversive odor-caffeine learning (Apostolopoulou et al., 2016; Choi et al., 2016). A current model, therefore, proposes that pharyngeal sensory neurons are activated upon food ingestion (Apostolopoulou et al., 2015; Miroschnikow et al., 2020; Maier et al., 2021). They instruct initiation or repression of food choice, feeding, and associative food learning through direct contact (Choi et al., 2020). In the case of sugars, sensory input from these neurons is combined with internal metabolic state signals, likely conveyed via the enteric system (Mishra et al., 2013; Hückesfeld et al., 2021; Schoofs et al., 2024).

Furthermore, many of the neurons of the DPS, DPO, and PPS are integrated into the pharyngeal organs of the adult, which contrasts with the general principle that adult sensory structures are born during metamorphosis and suggests that the pharyngeal organs may serve a conserved function throughout development (Gendre et al., 2004). Consistent with this, studies in adult *Drosophila* have demonstrated that the pharyngeal organs detect both chemical and physical properties of ingested food and contribute sensory feedback essential for coordinating the peristaltic motor program during unidirectional swallowing (Yang et al., 2021; Qin et al., 2024). Pharyngeal receptor neurons responsive to sugars facilitate the continuation of feeding bouts (LeDue et al., 2015; Yang et al., 2021), whereas those activated by bitter compounds or elevated salt concentrations robustly suppress food intake (Kim et al., 2017; Chen et al., 2021; Sang et al., 2024). In addition, pharyngeal mechanoreceptors relay information about food texture, enabling dynamic modulation of feeding rates to balance between complete food rejection and excessive consumption (Joseph et al., 2017; Yang et al., 2021; Qin et al., 2024). Together, these mechanisms coordinate nutrient homeostasis and the avoidance of harmful substances. Therefore, a better understanding of the building blocks that make up the pharyngeal sensory system would not only help to understand how insects organize food-guided behavior but also provide additional opportunities for pest control in *Drosophila* and insects in general.

Internal gustatory sensilla located in the cibarium and pharynx have been documented across multiple insect orders, including Diptera, Lepidoptera, Coleoptera, Hemiptera, Hymenoptera, Orthoptera, and Odonata (see Supplementary file 1; Ludwig, 1949; Ranade, 1967; Galić, 1971; Rice, 1973; McIver and Siemicki, 1981; Nayak and Singh, 1983; Lee and Craig, 1983; Kirby et al., 1984; Messchendorp et al., 1998; Glendinning et al., 2002; Gendre et al., 2004; Vegliante, 2005; Hückesfeld et al., 2010; Rebora et al., 2014; Baik and Carlson, 2020; Ortega-Insaurralde et al., 2024). Across these taxa, a common functional theme emerges: the evaluation of ingested material before and during food uptake, often through organs that combine gustatory and mechanosensory roles (Supplementary file 1). In *Drosophila* larvae and adults, the pharyngeal organs therefore likely reflect a conserved internal sensory strategy for assessing food quality, texture, and suitability, even though the number, morphology, and organization of sensilla differ between developmental stages (Gendre et al., 2004). Comparable roles are inferred in *Apis mellifera*, where the epi- and hypopharyngeal sensilla are thought to support nectar and pollen evaluation (Galić, 1971; de Brito Sanchez, 2011), and in lepidopteran larvae, where epipharyngeal or cibarial sensilla likely contribute to food acceptance and host-plant choice (Glendinning et al., 2002; Vegliante, 2005). In hemipterans such as *Rhodnius prolixus*, the pharyngeal organ is associated with food recognition in the context of blood feeding, indicating a similar requirement for internal evaluation in a distinct ecological niche (Ortega-Insaurralde et al., 2024). In other dipterans, the internal sensory system is adapted to different feeding ecologies: in *Aedes aegypti*, cibarial sensilla are linked to blood-feeding behavior in adult females (McIver and Siemicki, 1981; Lee and Craig, 1983; Baik and Carlson, 2020), which require a blood meal for egg production, whereas in larval *Musca domestica* and *Calliphora vicina,* mechanosensory roles of the pharyngeal organs were supposed, with limited or no direct evidence for strong gustatory control of feeding (Ludwig, 1949; Ranade, 1967; Hückesfeld et al., 2010). This comparison suggests that pharyngeal and cibarial organs can be broadly conserved as internal sensors for food evaluation, while their sensory focus shifts with diet, life history, and ecological niche. At the same time, the diversity of terms used for these structures likely reflects both conserved components of the insect oral sensory system and lineage-specific modifications of the mouthpart complex.

**Figure 1.**
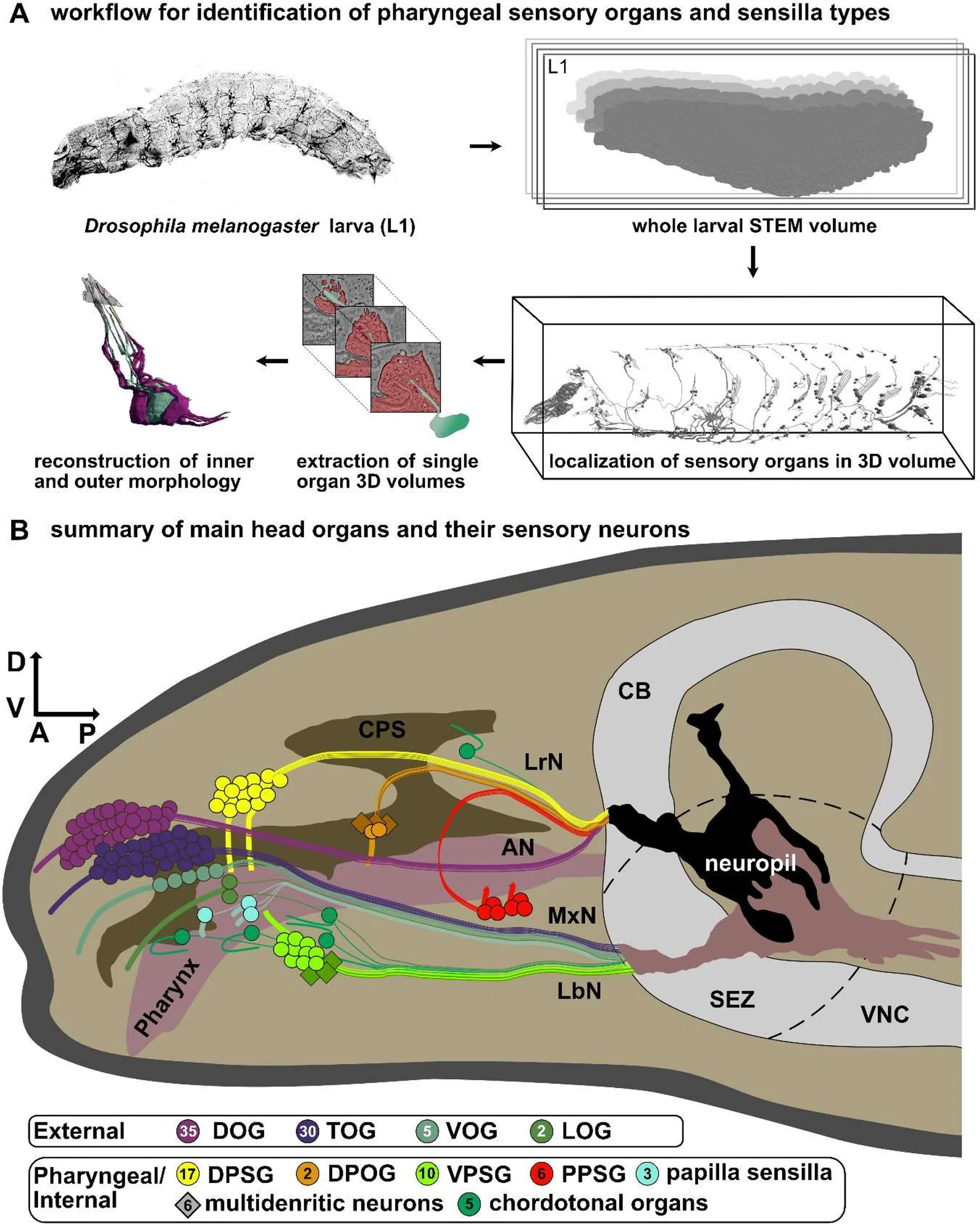
Localization and analysis of the cellular configuration of the larval pharyngeal sensilla. (**A**) Depiction of the workflow for identification of larval pharyngeal sense organs and sensilla types. A previously generated scanning transmission EM (STEM) volume of a whole first instar *D. melanogaster* larva (Peale et al., 2024) was used to localize all cells associated with the larval sensilla using CATMAID (Saalfeld et al., 2009). Smaller volumes were extracted from this volume to generate single organ or sensillum 3D volumes. These volumes were analyzed and manually reconstructed to describe and classify the types of pharyngeal sensilla and their corresponding sensory neurons. (**B**) Schematic drawing of the main external and pharyngeal organs of the larval head region. The projections via the main nerves into the subesophageal zone (SEZ) are visualized. Sensilla and sensory neurons were reconstructed from their distal ends to their ganglia. Axonal projections were not reconstructed. The numbers in the circles indicate the number of neurons in the respective ganglion. The pharynx (ocher), brain (gray), main external head organs, and pharyngeal organs are highlighted. These organs are the dorsal organ (DO; purple), the terminal organ (TO; blue), the ventral organ (VO; light blue), the labial organ (LO; turquoise), the dorsal pharyngeal sensilla (DPS; yellow), the dorsal pharyngeal organ (DPO; orange); ventral pharyngeal sensilla (VPS; bright green); and posterior pharyngeal sensilla (PPS; red). Abbreviations: D – dorsal; V – ventral; A – anterior; P – posterior; L1 – first instar; CB – central brain; SEZ – subesophageal zone; VNC – ventral nerve cord; AN – antennal nerve; MxN – maxillary nerve; LrN – labral nerve; LbN – labial nerve; CPS – cephalopharyngeal skeleton; DOG – dorsal organ ganglion; TOG - terminal organ ganglion; VOG – ventral organ ganglion; LOG – labial organ ganglion; DPSG – dorsal pharyngeal sensilla ganglion; DPOG – the dorsal pharyngeal organ ganglion; VPSG – ventral pharyngeal sensilla ganglion; PPSG – posterior pharyngeal sensilla ganglion.

In this study, we define the morphological types of pharyngeal sensilla based on their ultrastructure, utilizing scanning transmission electron microscopy (STEM) of a larval body volume, which enables the automated acquisition of serial sections at high resolution (Figure 1). The morphological characterization of the pharyngeal sensory structures is based on six criteria: (a) the presence of a terminal pore; (b) the presence of a sensillum shaft; (c) the presence of a cuticle tube; (d) the presence of a tubular body; (e) the number of dendrites; (f) lamellation of the thecogen cell (Table 1). We establish a comprehensive neuron-to-sensillum map for all four pharyngeal organs, identify previously undescribed neurons, and provide a precise classification and unified nomenclature. This work represents a significant step toward a complete analysis of the larval sensory system, providing new opportunities for investigating how *Drosophila* larvae process sensory information to navigate and interact with their environment. Furthermore, the detailed ultrastructural atlas established here provides a foundation for future comparative studies examining the morphological and functional organization of pharyngeal sensory systems in adult *Drosophila* and across other insect species, potentially revealing conserved design principles underlying internal taste sensation and feeding control.

**Table 1.** Structural properties of pharyngeal sensilla.

	VPS					DPS							DPO	PPS	
	P_1_	P_2_	P_mod_	T_1_	H_1_	T_1_	T_2_	T_3_	T_4_	T_5_	P/S_1_	P/S_2_	T_1_	T_1_	T_2_
Pore	+	+	–	+	–	+	+	+	+	+	–	–	+	+	+
Shaft	+	+	+	–	+	–	**–**	–	–	–	–	–	**–**	–	–
Tube	+	+	–	+	–	+	+	+	+	+	–	–	+	+	+
Tubular body	1	1	1	–	1	–	–	–	–	–	1	1	–	–	–
Dendrites	3	3	1	2	1	2	3	4	2	4	1	1	2	3	3
Lamellae	+	+	–	+	–	(+)	(+)	(+)	+	+	–	–	(+)	+	+

Abbreviations: VPS – ventral pharyngeal sensilla; DPS – dorsal pharyngeal sensilla; DPO – dorsal pharyngeal organ; PPS – posterior pharyngeal sensilla; P – papillum sensillum; P_mod_ – modified papillum sensillum; T – pit sensillum; H – hair sensillum; P/S – papilla/spot sensillum.

+ =structure present; (+) =structure weakly present; –  =structure not present.

## Results and discussion

### General

Pharyngeal sensilla share a common basic configuration typical of insect sensilla. They generally contain single or multiple bipolar Type I neurons (Zacharuk and Shields, 1991) surrounded by support cells (Schmidt and Berg, 1994; Keil, 1997; Klowden, 2007; Prelic et al., 2021). In contrast to multidendritic Type II neurons, these neurons extend only one dendrite from the cell body toward the outer cuticle or pharyngeal lumen (Klowden, 2007). The dendrites comprise inner and outer dendritic segments, separated by the ciliary constriction (Altner and Loftus, 1985). The outer segments are immersed in sensillum lymph, enclosed by the dendritic sheath and thecogen (sheath building) cell (Slifer, 1970; Altner and Prillinger, 1980; Zacharuk, 1980; Steinbrecht, 1984; Zacharuk and Shields, 1991). Specific structural features indicate sensory function. Mechanosensory Type I neurons typically display a tubular body – a dendritic tip densely packed with microtubules (Thurm, 1964; Keil, 1997). Chemosensory and especially gustatory sensilla are characterized by a terminal pore to the outside or the respective lumen (Slifer, 1970; Falk et al., 1976; Altner and Prillinger, 1980). This pore is usually formed by the cuticle tube, a structure thicker and less electron-dense than the dendritic sheath, first observed in *M. domestica* larval sensilla (Chu-Wang and Axtell, 1972). The origin of this structure remains unclear, but most likely, it originates from the dendritic sheath and is, therefore, built by the thecogen cell.

Our analysis utilized a STEM volume of a whole first instar *D. melanogaster* larva (Peale et al., 2024) to locate and reconstruct all sensory organs and their sensilla morphology (Figure 1A). While the external sensilla and the enteric sensory system were previously described using this dataset (Richter et al., 2024; Schoofs et al., 2024), we focus here on the sense organs along the pharynx, which primarily comprise gustatory sensilla that form part of the larval chemosensory system (Figure 1B). All pharyngeal organs and identified neurons are arranged bilaterally and thus organized in pairs, which we now describe in detail in the following.

### Ventral pharyngeal sensilla

**Figure 2.**
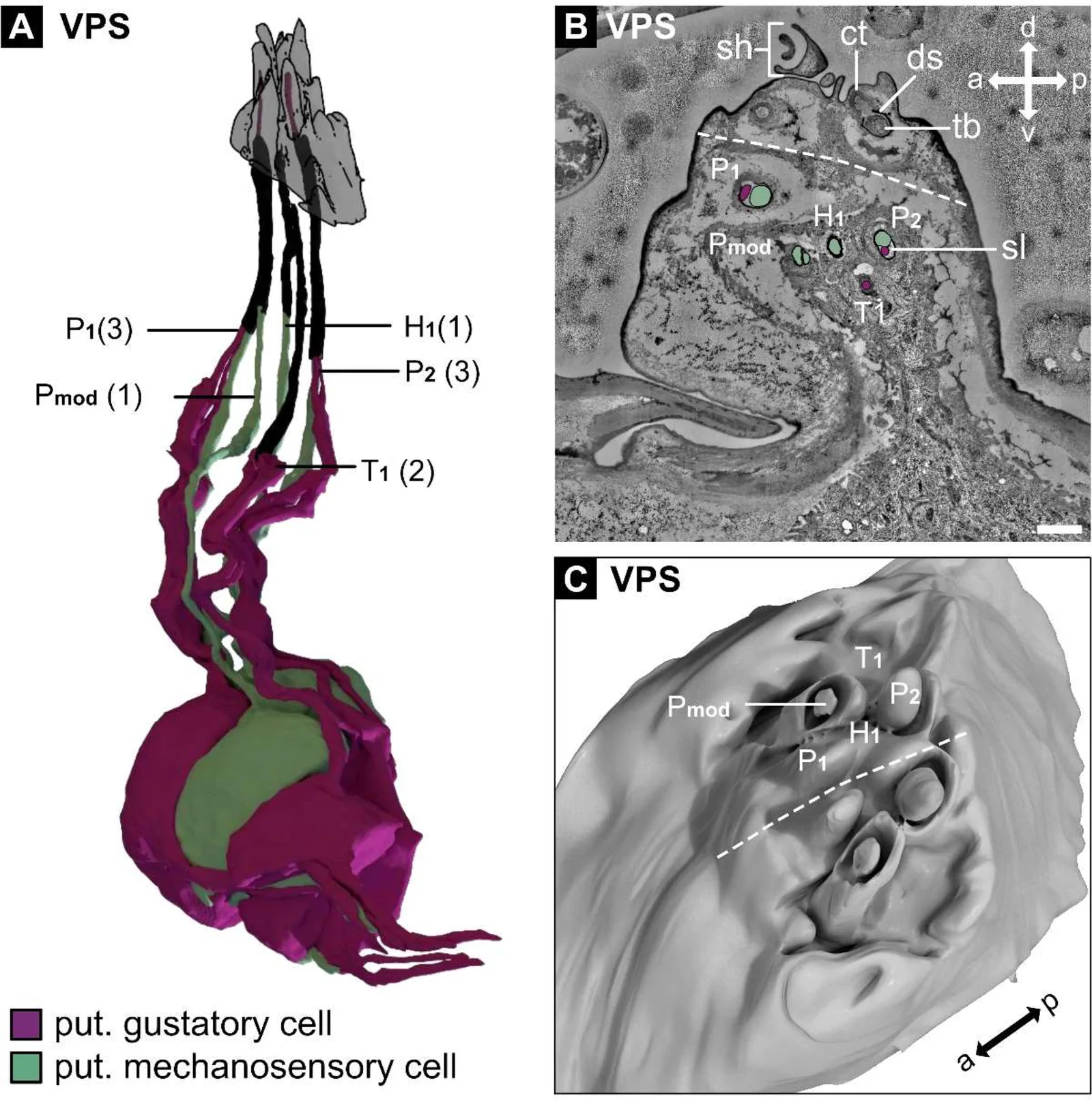
Main cellular configuration of the ventral pharyngeal sensilla (VPS). (**A**) 3D reconstruction of the sensory neurons innervating the VPS. The VPS is a bilaterally organized organ, but the left and right sides are fused. Only the reconstructions of the five sensilla on the left are shown. These are two papillum sensilla (P_1_ and P_2_), a modified papillum sensillum (P_mod_), a hair-like sensillum (H_1_), and a pit sensillum (T_1_). Ultrastructural features of the outer and inner morphology were used to classify the different sensilla. These sensilla resemble canonical sensilla types that can also be found in the terminal organ (see Figure 1A), the main external gustatory organ. They display different features like a terminal pore (pit sensillum – T) or a sensillum shaft (modified papillum sensillum – P_mod_; hair-like sensillum – H), or both (papillum sensillum – P). Mechanosensory cells are identified by their inner dendritic morphology, which contains a tubular body, a structure known to be important for mechanosensation. For a detailed description of the single VPS sensilla, see Figure 2—figure supplements 1–5. Nomenclature for sensilla was adapted from previous work (Chu-Wang and Axtell, 1972; Rist and Thum, 2017). The number of neurons innervating the sensilla is indicated in brackets. (**B**) STEM section of the VPS showing the organ’s midline (dashed line). The sensilla of the right side are highlighted, and the innervating neurons are color-coded for their putative sensory function (purple – gustatory; green – mechanosensory). (**C**) 3D reconstruction of the outer morphology of the VPS. The position of the sensilla is indicated for the right side of the fused organ. Unlike the other pharyngeal sensilla (see Figures 3 and 4), the VPS display prominent outer structures that extend into the pharyngeal cavity. Abbreviations: d – dorsal; v – ventral; a – anterior; p – posterior; P – papillum sensillum; P_mod_ –modified papillum sensillum; H – hair sensillum; T – pit sensillum. Scale bars: B: 1 µm.

In contrast to other pharyngeal sensilla, the VPS displays prominent outer structures that extend into the pharyngeal lumen (Figure 2). It is a bilateral organ, but the left and right sides are fused (Figure 2A–C). Each side contains five individual sensilla innervated by a total of 10 Type I neurons per side (Figure 2A). These sensilla resemble sensilla types found in the TO and other external sensilla. It consists of two papillum sensilla (P_1_ and P_2_) (Figure 2—figure supplement 1 and 2), one modified papillum sensillum (P_mod_) (Figure 2—figure supplement 3), a hair sensillum (H_1_) (Figure 2—figure supplement 4), and a pit sensillum (T_1_) (Figure 2—figure supplement 5). All VPS sensilla display the standard set of support cells that enclose the sensory neurons and the sensillum lymph (Figure 2—figure supplements 1–5F; Figure 2—figure supplement 5E), but the thecogen cells show different degrees of lamellation (Table 1; Figure 2—figure supplement 1 and 2H; Figure 2—figure supplements 3–5G). The papillum sensilla P_1_ and P_2_ display a shaft with a terminal pore to the pharyngeal lumen (Figure 2—figure supplement 1 and 2C) and a cuticle tube (ct) (Figure 2—figure supplement 1 and 2D) that connects with the dendritic sheath (Figure 2—figure supplement 1 and 2E). Both are innervated by three dendrites each, two that protrude into the pore and, therefore, most likely serve a gustatory function. The third one ends with a tubular body (Figure 2—figure supplement 1 and 2E) and, therefore, most likely serves a mechanosensory function. P_mod_ displays a shaft that forms a cylindrical portion that encircles a bud-like structure (Figure 2—figure supplement 3C). It has a single dendrite that ends with a tubular body below the bud. The dendritic sheath remains intact, and a pore is absent. P_mod_ most likely serves a mechanosensory function. H_1_ is internally built in a similar configuration as the P_mod_, containing a tubular body and no pore to the lumen. Only the sensillum shaft is built differently (Figure 2—figure supplement 4C) and resembles the hair sensilla found in the larval body segments. The different outer morphologies may induce different mechanisms in mechanotransduction or, at least, different stimulus strengths needed (Figure 2—figure supplement 3 and 4D, E). T_1_ resembles a pit sensillum, containing a pore to the lumen and a cuticle tube that connects to the dendritic sheath (Figure 2—figure supplement 5C, D). The pore channel is innervated by two, most likely gustatory neurons. All cell bodies of neurons innervating the VPS lie in the VPS ganglion (VPSG) (Figure 2, Figure 2—figure supplements 1–5I). Furthermore, three multidendritic Type II neurons are present in the VPSG (Figure 2—figure supplement 3I). All VPSG neurons send axons to the subesophageal zone (SEZ) via the labial nerve (LbN).

**Figure 2—figure supplement 1.**
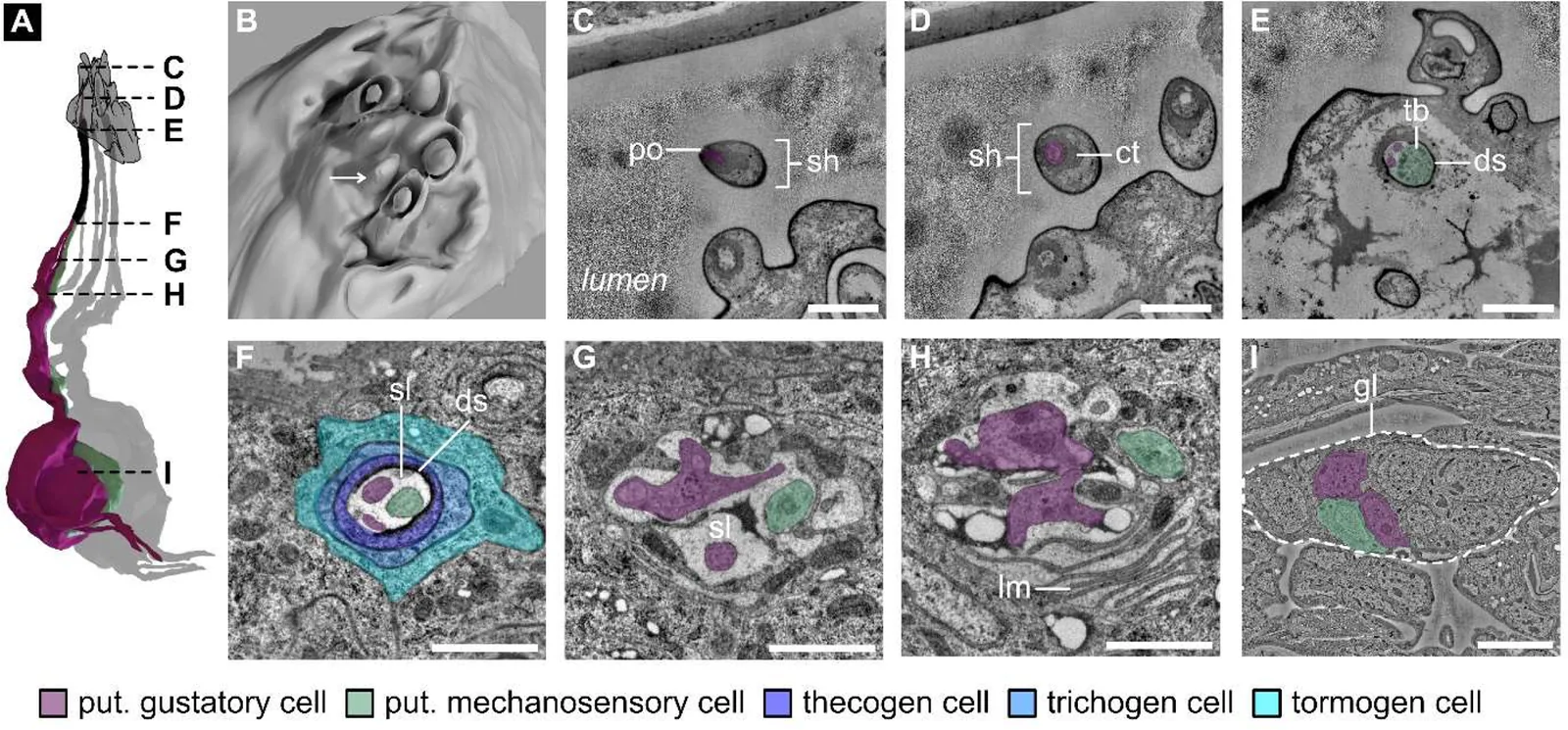
VPS-P_1_. Internal morphology of the papillum sensillum 1 (P_1_) of the ventral pharyngeal sensilla (VPS). (**A**) 3D reconstruction of the sensory neurons innervating the P_1_ sensillum. Three sensory neurons innervate P_1_. (**B**) 3D reconstruction of the outer morphology of the VPS. The arrow indicates the location of the P_1_ sensillum. (**C–I**) Serial scanning transmission electron microscopy (STEM) sections of the P_1_ sensillum. (**C, D**) The sensillum shaft is visible. The terminal pore is innervated by sensory neurons (purple). (**E**) Further proximal, all three cilia are visible. Two neurons (purple) are innervating the terminal pore, and one neuron (turquoise) ends at the base of the pore with a tubular body. A dendritic sheath is present. (**F**) The three dendrites are bathed in the sensillum lymph and enclosed by the dendritic sheath. The thecogen, trichogen, and tormogen support cells are highlighted. (**G**) The three dendrites are bathed in the sensillum lymph, but the dendritic sheath is absent at this level. (**H**) The sheath cell is highly compartmented and lamellated at this level. (**I**) Section of the VPS ganglion. The cell bodies of the three P_1_ neurons are visible. Abbreviations: po – pore; sh – shaft; ct – cuticle tube; ds – dendritic sheath; tb – tubular body; sl – sensillum lymph; lm – lamellae. Scale bars: C–H: 1 µm; I: 5 µm.

**Figure 2—figure supplement 2.**
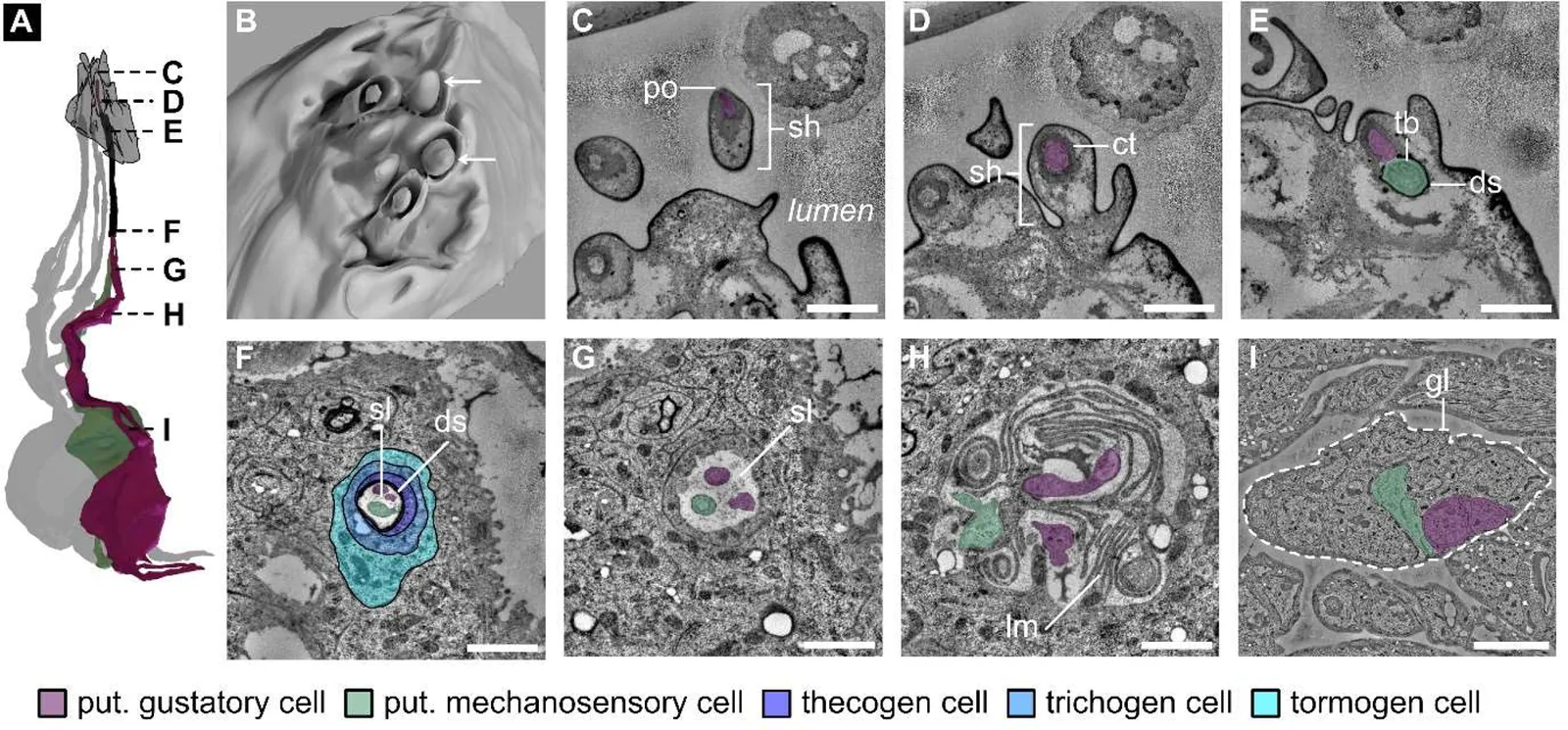
VPS-P_2_. Internal morphology of the papillum sensillum 2 (P_2_) of the ventral pharyngeal sensilla (VPS). (**A**) 3D reconstruction of the sensory neurons innervating the P_2_ sensillum. Three sensory neurons innervate P_2_. (**B**) 3D reconstruction of the outer morphology of the VPS. The arrow indicates the location of the P_2_ sensillum. (**C–I**) Serial scanning transmission electron microscopy (STEM) sections of the P_2_ sensillum. (**C, D**) The sensillum shaft is visible. The terminal pore is innervated by sensory neurons (purple). (**E**) Further proximal, all three cilia are visible. Two neurons (purple) are innervating the terminal pore, and one neuron (turquoise) ends at the base of the pore with a tubular body. A dendritic sheath is present. (**F**) The three dendrites are bathed in the sensillum lymph and enclosed by the dendritic sheath. The thecogen, trichogen, and tormogen support cells are highlighted. (**G**) The three dendrites are bathed in the sensillum lymph, but the dendritic sheath is absent at this level. (**H**) The sheath cell is highly compartmented and lamellated at this level. (**I**) Section of the VPS ganglion. The cell bodies of the three P_2_ neurons are visible. Abbreviations: po – pore; sh - shaft; ct – cuticle tube; ds – dendritic sheath; tb – tubular body; sl – sensillum lymph; lm – lamellae; gl – ganglion. Scale bars: C–H: 1 µm; I: 5 µm.

**Figure 2—figure supplement 3.**
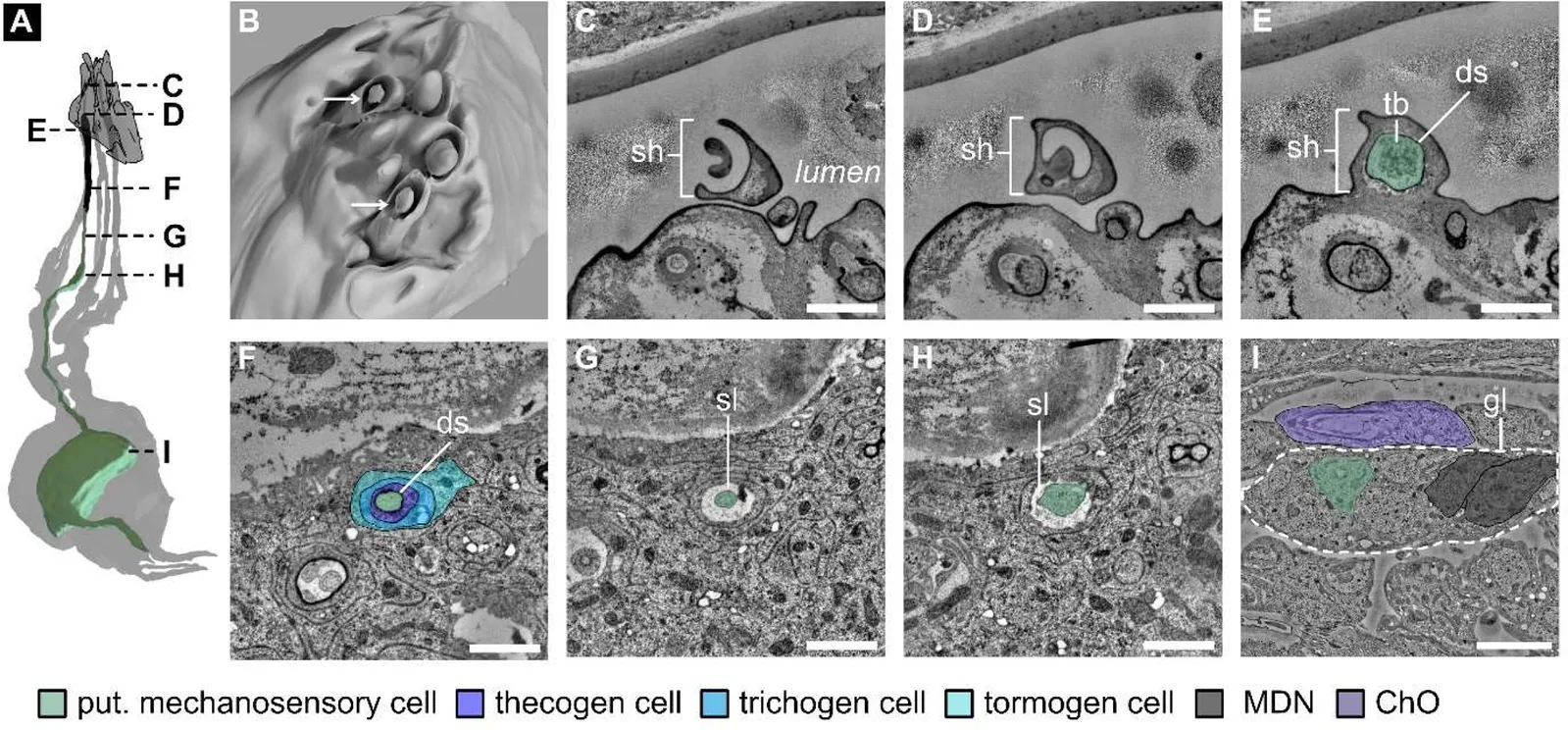
VPS-P_mod_. Internal morphology of the modified papillum sensillum (P_mod_) of the ventral pharyngeal sensilla (VPS). (**A**) 3D reconstruction of the P_mod_ sensillum. A single sensory neuron innervates P_mod_. (**B**) 3D reconstruction of the outer morphology of the VPS. The arrow indicates the location of the P_mod_ sensillum. (**C–I**) Serial scanning transmission electron microscopy (STEM) sections of the P_mod_ sensillum. (**C, D**) The sensillum shaft is visible. Its shaft forms a cylindrical portion that encircles a bud-like structure. The bud lacks a terminal pore. (**E**) Further proximal, the neuron (turquoise) at the base of the shaft is visible. The neuron ends with a tubular body. A dendritic sheath is present. (**F**) The dendrite is entirely enclosed by the dendritic sheath. The thecogen, trichogen, and tormogen support cells are highlighted. (**G, H**) The dendrite is bathed in the sensillum lymph, but the dendritic sheath is absent at this level. The sheath cell is not compartmented and lamellated. (**I**) Section of the VPS ganglion (VPSG). The cell body of the P_mod_ neuron is visible. Additionally, three cell bodies of multidendritic sensory neurons (Type II neurons) lie in the VPSG (two cell bodies and one dendrite visible in this section). A heterodynal chordotonal organ lies close to the VPSG. Abbreviations: sh – shaft; tb – tubular body; ds – dendritic sheath; sl – sensillum lymph; gl – ganglion; MDN – multidendritic neuron; ChO – chordotonal organ. Scale bars: C–H: 1 µm; I: 5 µm.

**Figure 2—figure supplement 4.**
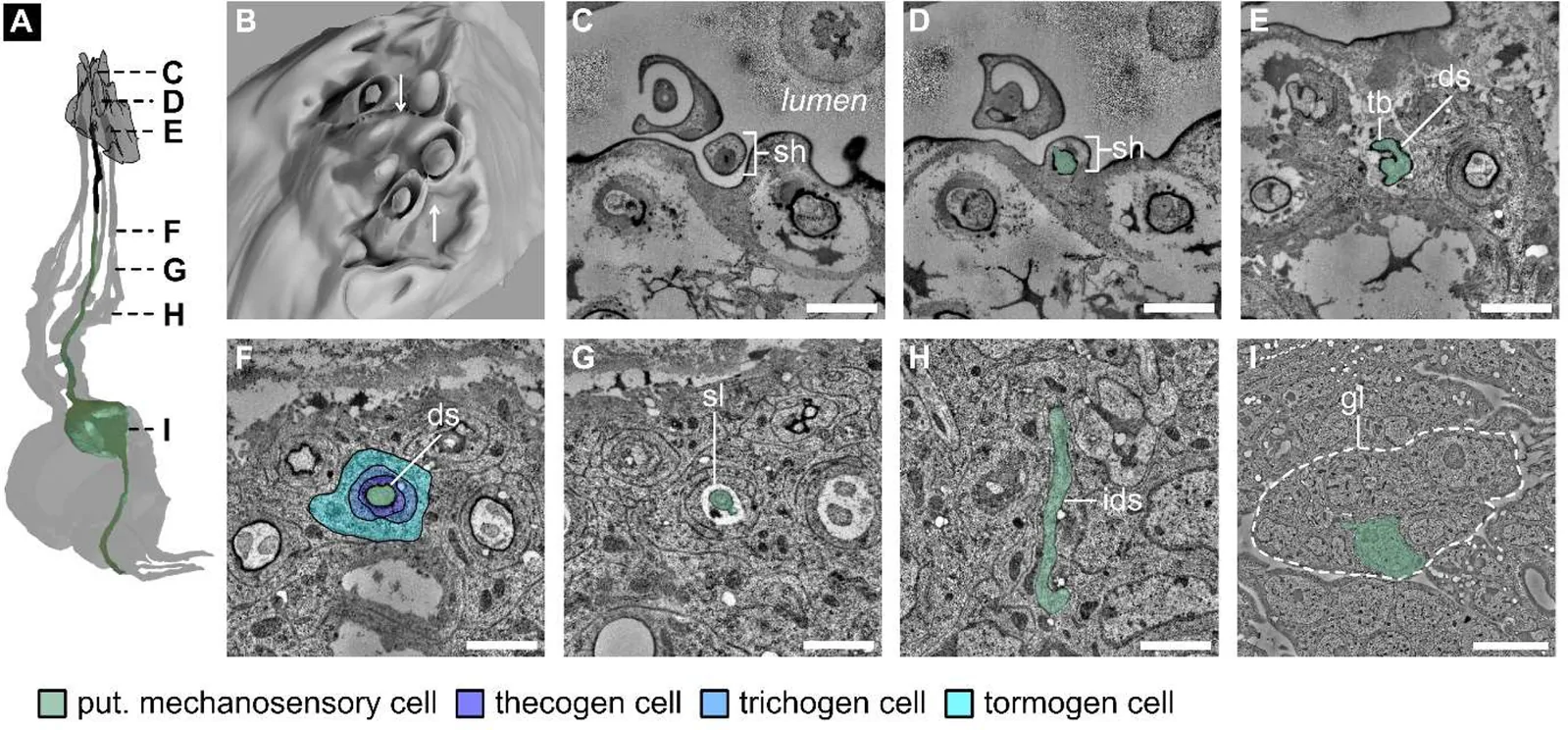
VPS-H_1_. Internal morphology of the hair-like sensillum 1 (H_1_) of the ventral pharyngeal sensilla (VPS). (**A**) 3D reconstruction of the H_1_ sensillum. A single sensory neuron innervates H_1_. (**B**) 3D reconstruction of the outer morphology of the VPS. The arrow indicates the location of the H_1_ sensillum. (**C–I**) Serial scanning transmission electron microscopy (STEM) sections of the H_1_ sensillum. (**C, D**) The sensillum shaft is visible. Its shaft forms a hair-like structure. The hair lacks a terminal pore. Externally, the shaft is hardly visible as the other prominent VPS sensilla mask it. (**D, E**) The neuron (turquoise) at the base of the shaft is visible. The neuron ends with a tubular body. A dendritic sheath is present. (**F**) The dendrite is entirely enclosed by the dendritic sheath. (**G**) The dendrite is bathed in the sensillum lymph, but the dendritic sheath is absent at this level. The thecogen, trichogen, and tormogen support cells are highlighted. (**H**) The sheath cell is not compartmented and lamellated. (**I**) Section of the VPS ganglion. The cell body of the H_1_ neuron is visible. Abbreviations: sh – shaft; ds – dendritic sheath; tb – tubular body; sl – sensillum lymph; ids – inner dendritic segment; gl – ganglion. Scale bars: C–H: 1 µm; I: 5 µm.

**Figure 2—figure supplement 5.**
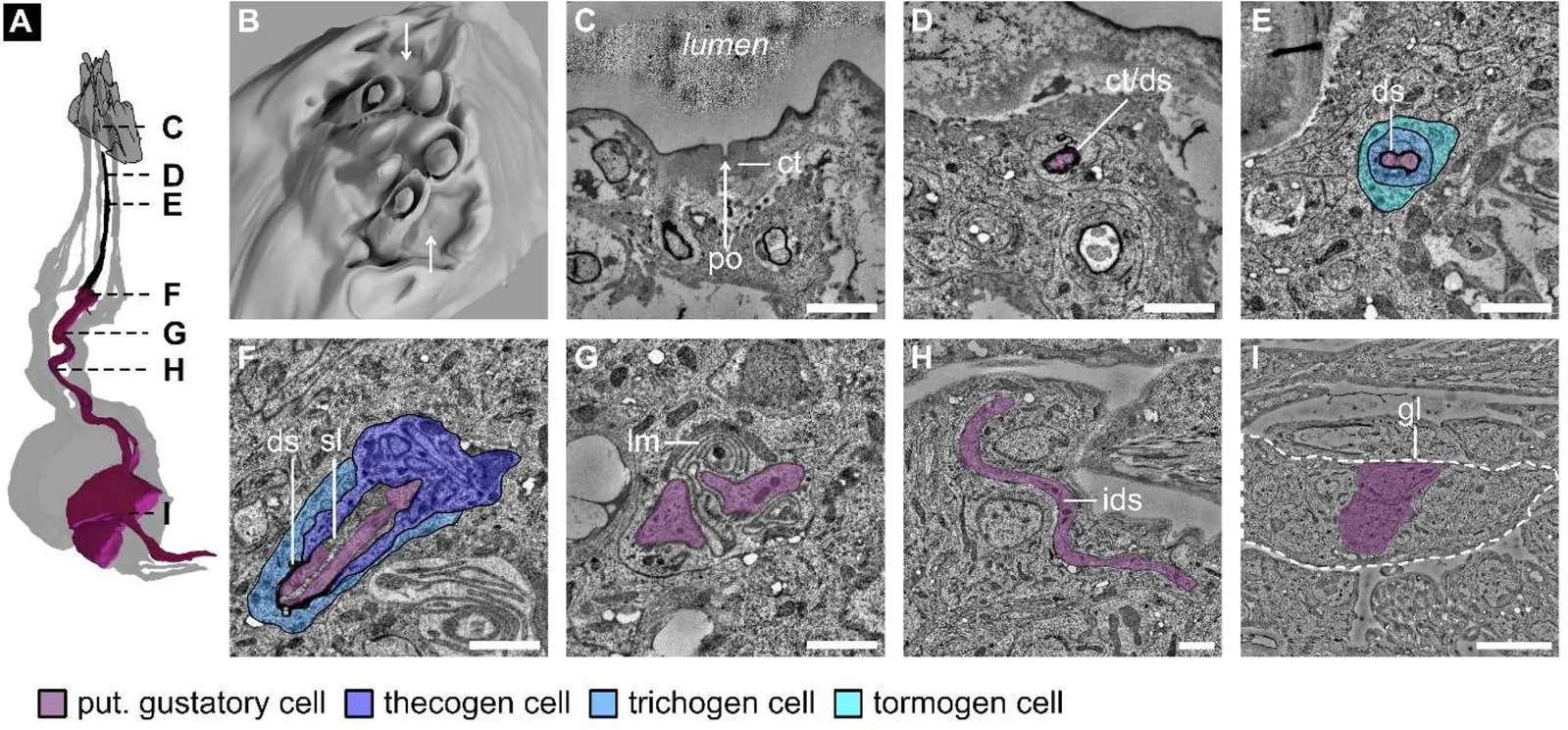
VPS-T_1_. Internal morphology of the pit sensillum 1 (**T_1_**) of the ventral pharyngeal sensilla (VPS). (**A**) 3D reconstruction of the T_1_ sensillum. Two sensory neurons innervate T_1_. (**B**) 3D reconstruction of the outer morphology of the VPS. The arrow indicates the location of the T_1_ sensillum. (**C–I**) Serial scanning transmission electron microscopy (STEM) sections of the T_1_ sensillum. (**C**) The terminal pore is visible. A cuticle tube connects to the lumen through a pore and is innervated by the dendrites. (**D**) The cuticle tube connects with the dendritic sheath. (**E**) The two ciliary structures (purple) are visible. A dendritic sheath is present. The trichogen and tormogen support cells are highlighted. (**F**) The tips of the dendrites are enclosed by the dendritic sheath, and the proximal parts are bathed in the sensillum lymph enclosed by the sheath cell. The thecogen and trichogen support cells are highlighted. (**G**) The dendrite is bathed in the sensillum lymph, but the dendritic sheath is absent at this level. The sheath cell is lamellated. (**H**) Inner dendritic segment of one sensory cell (**I**) Section of the VPS ganglion. The cell bodies of the T_1_ neurons are visible. Abbreviations: po – pore; ct – cuticle tube; ds – dendritic sheath; sl – sensillum lymph; lm – lamellae; ids – inner dendritic segment; gl – ganglion. Scale bars: C–H: 1 µm; I: 5 µm.

### Dorsal pharyngeal sensilla

**Figure 3.**
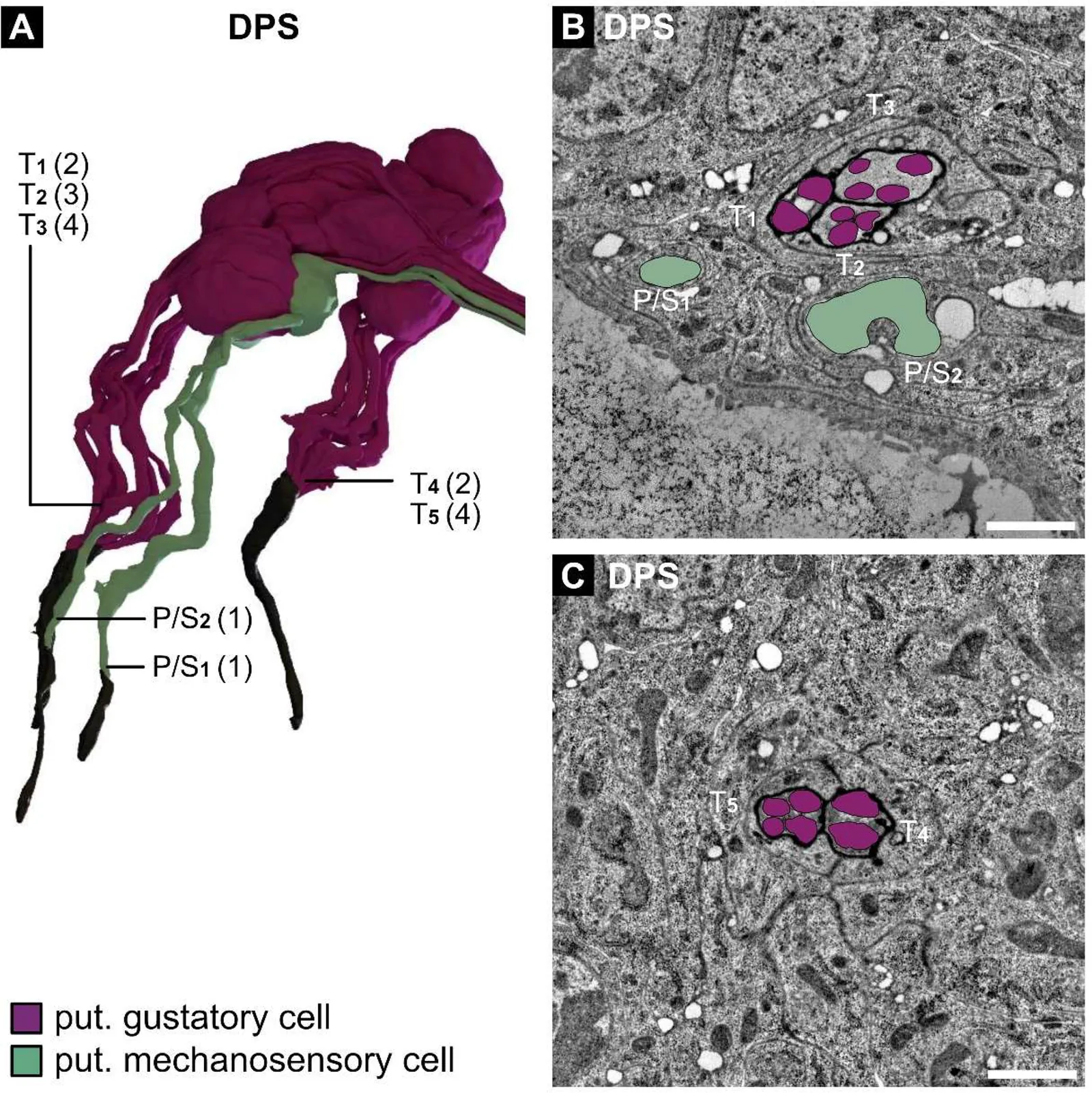
Main cellular configuration of the dorsal pharyngeal sensilla (DPS). (**A**) 3D reconstruction of the sensory neurons innervating the DPS. The DPS is a bilaterally organized organ; only the reconstructions of the seven sensilla on the left are shown. These are two papilla/spot sensilla (P/S_1_ and P/S_2_) and five pit sensilla (T_1_ – T_5_). From the ganglion, the sensilla expand to an anterior and a posterior location. In the anterior group, we find three pit sensilla (T_1_, T_2_, and T_3_) and two papilla/spot sensilla (P/S_1_ and P/S_2_), the latter of which is located in a deep channel. The pit sensilla display an idiosyncrasy as they share their terminal pore into the pharyngeal lumen. The posterior group consists of two pit sensilla (T_4_ and T_5_), which share a terminal pore, too. Ultrastructural features of the outer and inner morphology were used to classify the sensilla. For a detailed description of the single DPS sensilla, see Figure 3—figure supplements 1–7. The nomenclature for sensilla was adapted from previous work (Chu-Wang and Axtell, 1972; Rist and Thum, 2017). The number of neurons innervating the sensilla is indicated in brackets. (**B, C**) Scanning transmission electron microscopy (STEM) section of the anterior group (**B**) and posterior group (**C**) of the DPS. The sensory neurons innervating the sensilla are highlighted and color-coded for their putative sensory function (purple – gustatory; green – mechanosensory). Abbreviations: d – dorsal; v – ventral; a – anterior, p – posterior; P/S – papilla/spot sensillum; T – pit sensillum. Scale bars: B: 1 µm; C: 1 µm.

The DPS is a bilaterally organized organ that contains seven sensilla on each side, innervated by a total of 17 Type I neurons per side. From the ganglion, the neurons extend their dendrites toward anterior and posterior locations in the pharyngeal cavity (Figure 3A–C; Figure 3—figure supplements 1–7A, B). In the anterior location, we found three pit sensilla (T_1_, T_2_, and T_3_) and two papilla/spot sensilla (P/S_1_ and P/S_2_), the latter located in a deep depression (Figure 3—figure supplement 2B). Papilla and spot sensilla display the same morphology, but the former are termed spot sensilla when found within a compound sensillum (Rist and Thum, 2017) and papilla sensilla when occurring as a single sensillum (Dambly-Chaudière and Ghysen, 1986; Richter et al., 2024). In the posterior location, we identified two pit sensilla (T_4_ and T_5_). In contrast to the pit sensilla of other larval gustatory organs, the DPS pit sensilla of the anterior group (T_1_, T_2_, and T_3_) and the posterior group (T_4_ and T_5_) share a cuticle tube and a terminal pore that opens into the pharyngeal lumen (Figure 3—figure supplements 3–7B, C).

**Figure 3—figure supplement 1.**
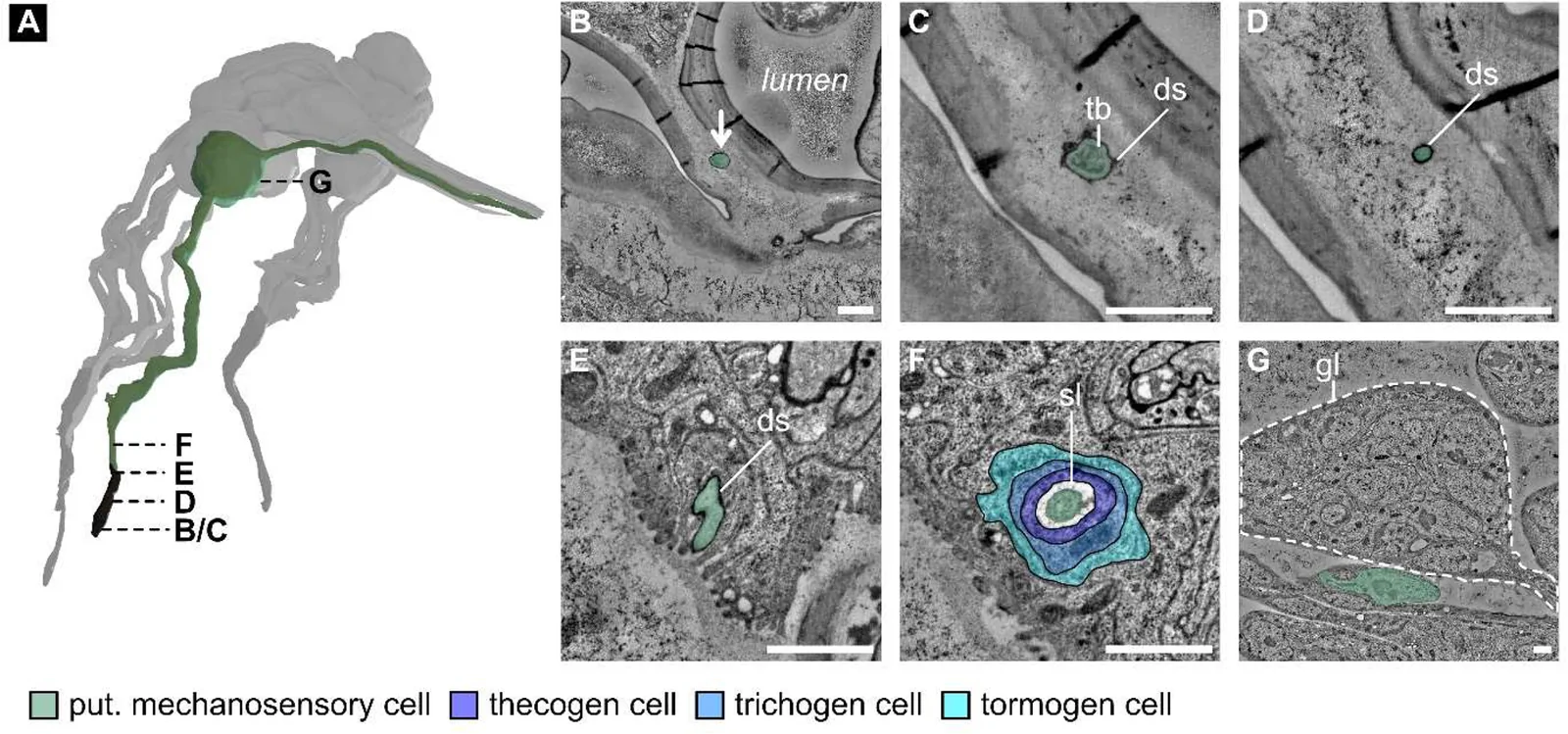
DPS-P/S_1_. Internal morphology of the papilla/spot sensillum 1 (P/S_1_) of the dorsal pharyngeal sensilla (DPS). (**A**) 3D reconstruction of the P/S_1_ sensillum. A single sensory neuron (turquoise) innervates P/S1. (**B–G**) Serial scanning transmission electron microscopy (STEM) sections of the P/S_1_ sensillum. (**B**) The P/S1 sensillum sits at the anterior end of the pharyngeal cavity (ph). (**C**) Its dendrite exhibits a tubular body at the tip. A dendritic sheath is present. The sensillum lacks a terminal pore. (**D**) Further proximal, the dendrite tapers down and thickens again (**E**). The dendrite is entirely enclosed by the dendritic sheath. (**F**) The dendrite is bathed in the sensillum lymph, but the dendritic sheath is absent at this level. The thecogen, trichogen, and tormogen support cells are highlighted. (**G**) Section of the VPS ganglion. The cell body of the P/S_1_ neuron is visible but lies beside the ganglion. Abbreviations: ds – dendritic sheath; tb – tubular body; sl – sensillum lymph; gl – ganglion. Scale bars: B–G: 1 µm.

**Figure 3—figure supplement 2.**
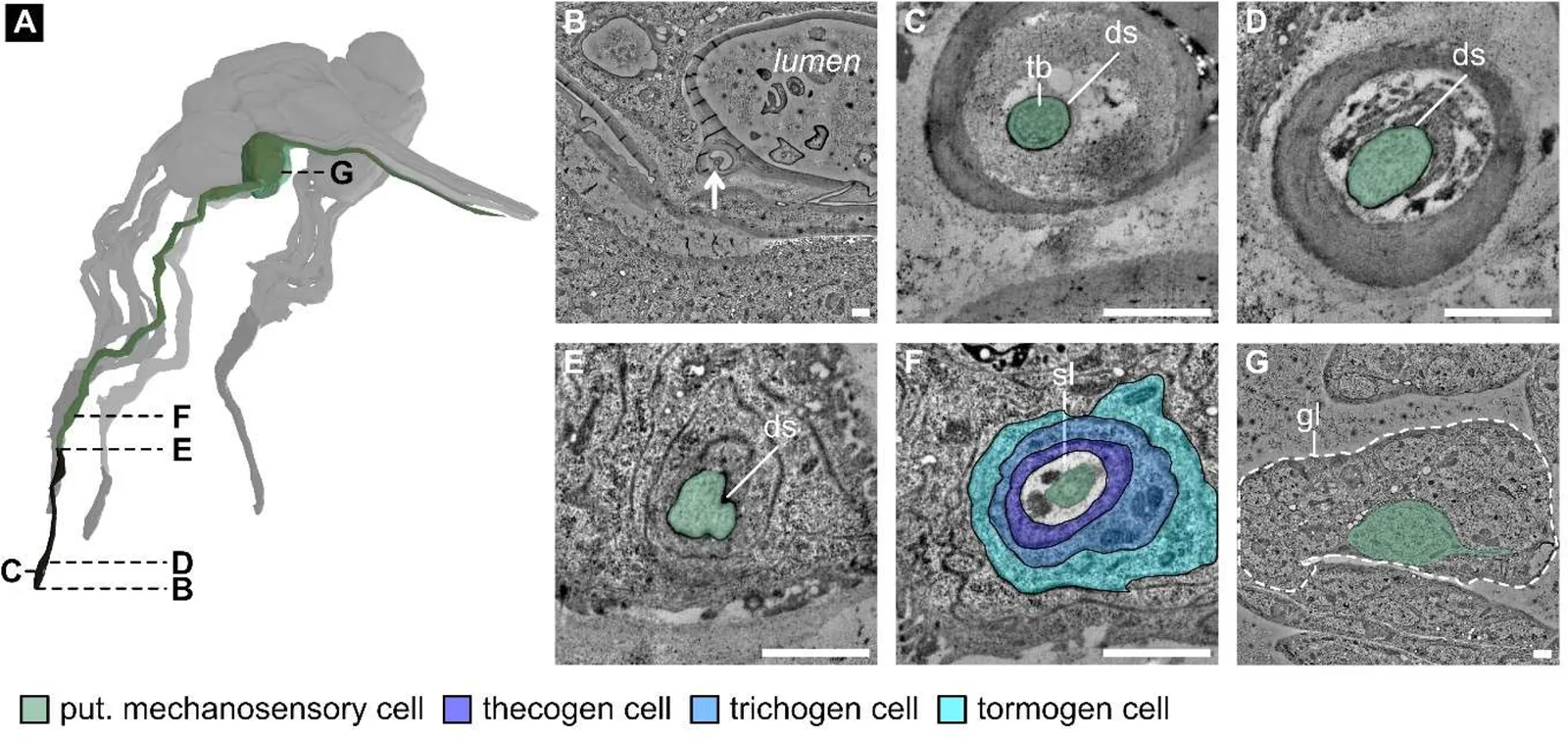
DPS-P/S2. Internal morphology of the papilla/spot sensillum 2 (P/S_2_) of the dorsal pharyngeal sensilla (DPS). (**A**) 3D reconstruction of the P/S_2_ sensillum. A single sensory neuron (turquoise) innervates S2. (**B–G**) Serial scanning transmission electron microscopy (STEM) sections of the P/S_2_ sensillum. (**B**) The P/S_2_ sensillum sits at the anterior end of the pharyngeal cavity (ph) in a deep ‘cuticle’ channel (2,5 µm). (**C**) Its dendrite exhibits a tubular body at the tip. A dendritic sheath is present. The sensillum lacks a terminal pore. (**D**) Further proximal, the dendrite thickens (**E**). The dendritic sheath entirely encloses the dendrite. (**F**) The dendrite is bathed in the sensillum lymph, but the dendritic sheath is absent at this level. The thecogen, trichogen, and tormogen support cells are highlighted. (**G**) Section of the VPS ganglion. The cell body of the P/S_2_ neuron is visible and lies inside the ganglion. Abbreviations: ds – dendritic sheath; tb – tubular body; sl – sensillum lymph; gl – ganglion. Scale bars: B–G: 1 µm.

**Figure 3—figure supplement 3.**
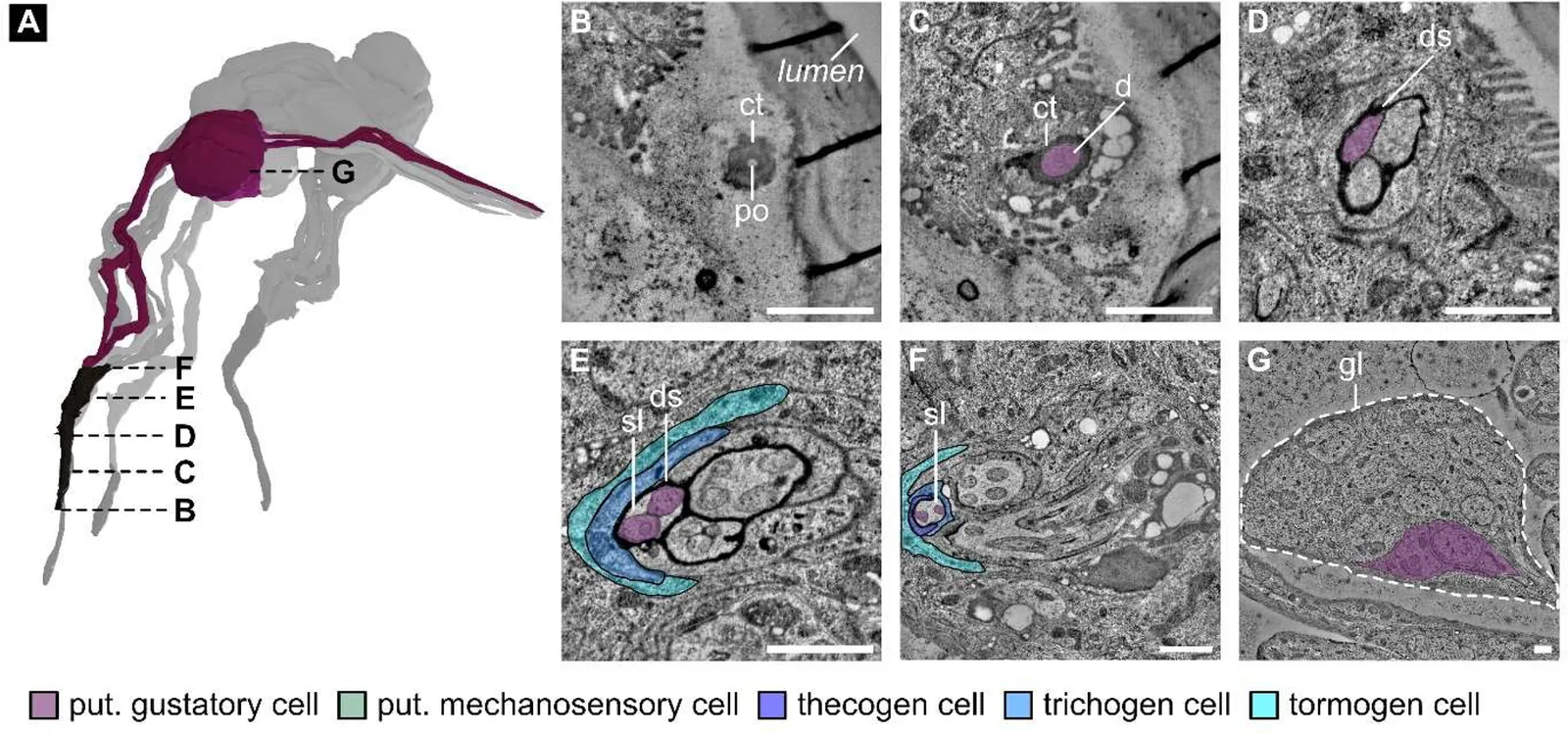
DPS-T_1_. Internal morphology of the pit sensillum 1 (T_1_) of the dorsal pharyngeal sensilla (DPS). (**A**) 3D reconstruction of the T_1_ sensillum. Two sensory neurons (purple) innervate T_1_. The sensillum shares a terminal pore to the pharyngeal lumen with pit sensilla T_2_ and T_3_ (see Figure 3—figure supplements 4 and 5). (**B–G**) Serial scanning transmission electron microscopy (STEM) sections of the T_1_ sensillum. (**B**) The T_1_ sensillum sits in a small pore at the anterior end of the pharyngeal cavity. (**C**) Its dendrites extend into the pore through the cuticle tube, together with the T_2_ and T_3_ dendrites. (**D**) Further proximal, the dendritic sheaths separate the dendrites of T_1_ – T_3_ from each other. (**E**) The dendrites’ ciliary structures are visible and entirely enclosed by the dendritic sheath(s). The trichogen and tormogen support cells are visible. (**F**) The dendrites are bathed in the sensillum lymph, but the dendritic sheath disintegrates at this level. The thecogen, trichogen, and tormogen support cells are visible. (**G**) Section of the VPS ganglion. The cell bodies of the T_1_ neurons are visible and lie inside the ganglion. Abbreviations: po – pore; ct – cuticle tube; d – dendrites; ds – dendritic sheath; sl – sensillum lymph; lm – lamellae; gl – ganglion. Scale bars: B–G: 1 µm.

**Figure 3—figure supplement 4.**
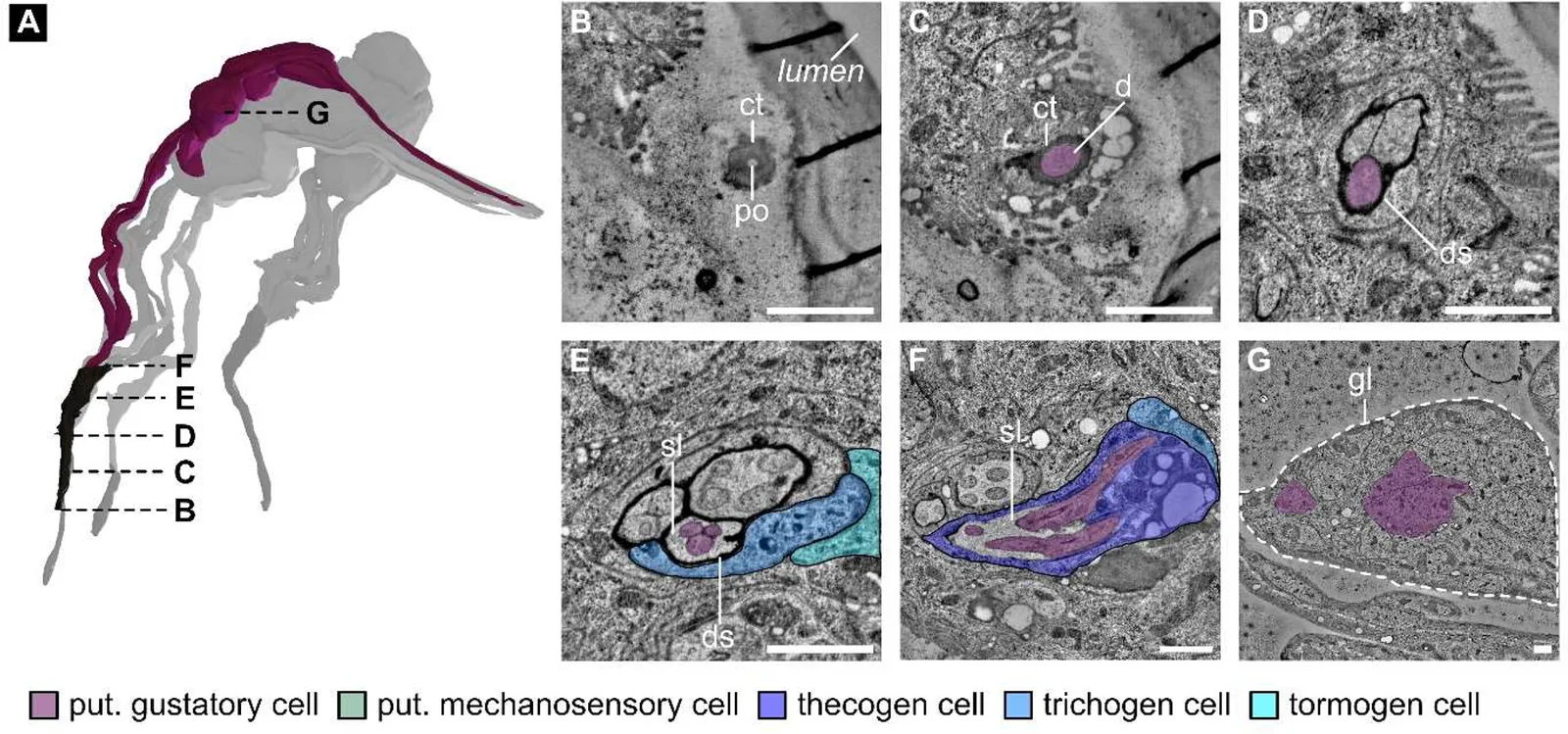
DPS-T_2_. Internal morphology of the pit sensillum 2 (T_2_) of the dorsal pharyngeal sensilla (DPS). (**A**) 3D reconstruction of the T_2_ sensillum. Three sensory neurons (purple) innervate T_2_. The sensillum shares a terminal pore to the pharyngeal lumen with pit sensilla T_1_ and T_3_ (see Figure 3—figure supplements 3 and 5). (**B–G**) Serial scanning transmission electron microscopy (STEM) sections of the T_2_ sensillum. (**B**) The T_2_ sensillum sits in a small pore at the anterior end of the pharyngeal cavity (ph). (**C**) Its dendrites extend into the pore through the cuticle channel, together with the T_1_ and T_3_ dendrites. (**D**) Further proximal, a dendritic sheath appears that separates the dendrites of T_1_ – T_3_ from each other. (**E**) The dendrites’ ciliary structures are visible and entirely enclosed by the dendritic sheath(s). The trichogen and tormogen support cells are visible. (**F**) The dendrites are bathed in the sensillum lymph, but the dendritic sheath is entirely absent at this level. The thecogen and trichogen support cells are visible. (**G**) Section of the VPS ganglion. The cell bodies of the T_2_ neurons are visible and lie inside the ganglion. Abbreviations: po – pore; ct – cuticle tube; d – dendrites; ds – dendritic sheath; sl – sensillum lymph; lm – lamellae; gl – ganglion. Scale bars: B–G: 1 µm.

**Figure 3—figure supplement 5.**
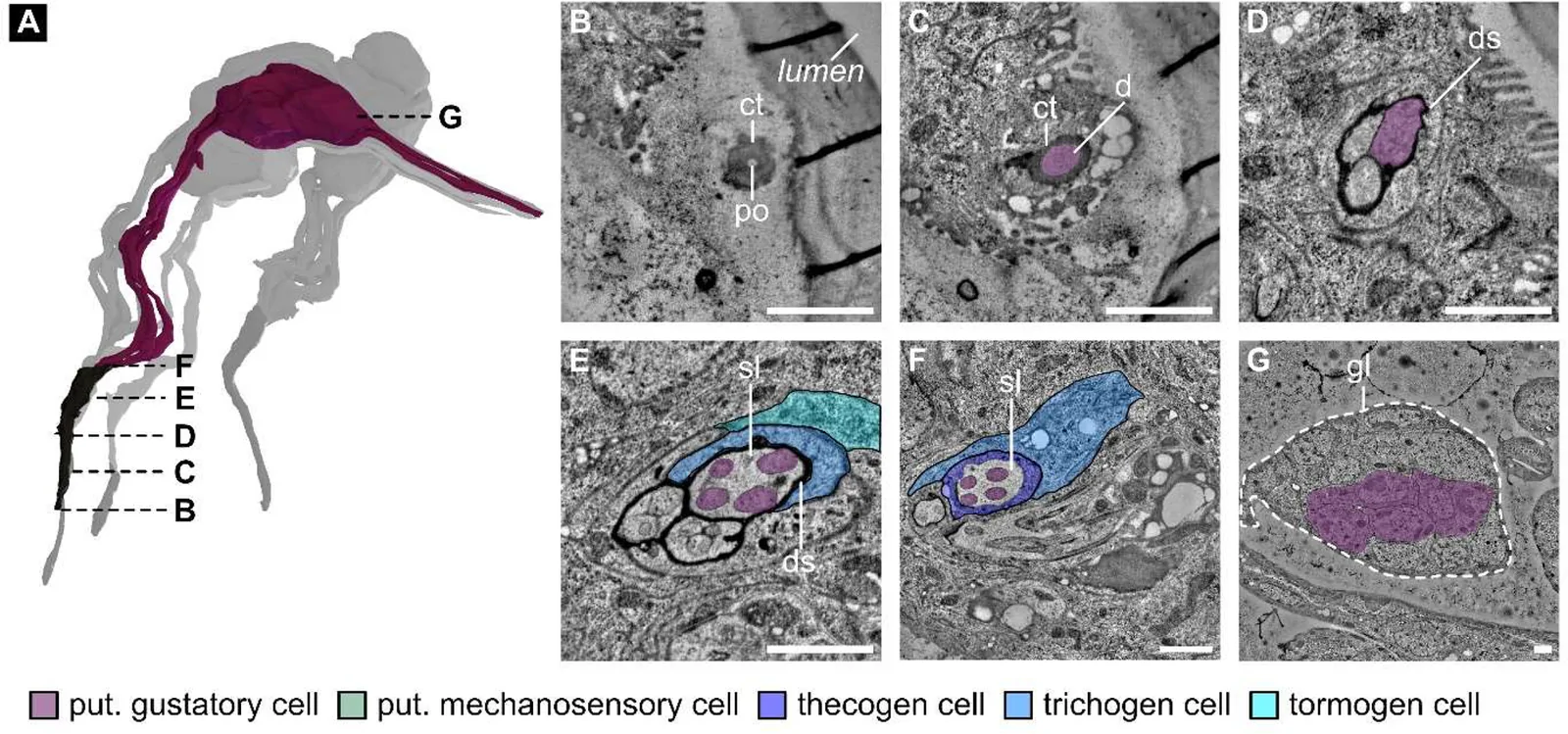
DPS-T_3_. Internal morphology of the pit sensillum 3 (T_3_) of the dorsal pharyngeal sensilla (DPS). (**A**) 3D reconstruction of the T_3_ sensillum. Four sensory neurons (purple) innervate T_3_. The sensillum shares a terminal pore to the pharyngeal lumen with pit sensilla T_1_ and T_2_ (see Figure 3—figure supplements 3 and 4). (**B–G**) Serial scanning transmission electron microscopy (STEM) sections of the T_3_ sensillum. (**B**) The T_3_ sensillum sits in a small pore at the anterior end of the pharyngeal cavity (ph). (**C**) Its dendrites extend into the pore through the cuticle channel, together with the T_1_ and T_2_ dendrites. (**D**) Further proximal, a dendritic sheath appears that separates the dendrites of T_1_ – T_3_ from each other. (**E**) The dendrites’ ciliary structures are visible and entirely enclosed by the dendritic sheath(s). The trichogen and tormogen support cells are visible. (**F**) The dendrites are bathed in the sensillum lymph, but the dendritic sheath is entirely absent at this level. The thecogen and trichogen support cells are visible. (**G**) Section of the VPS ganglion. The cell bodies of the T_3_ neurons are visible and lie inside the ganglion. Abbreviations: po – pore; ct – cuticle tube; d – dendrites; ds – dendritic sheath; sl – sensillum lymph; lm – lamellae; gl – ganglion. Scale bars: B–G: 1 µm.

**Figure 3—figure supplement 6.**
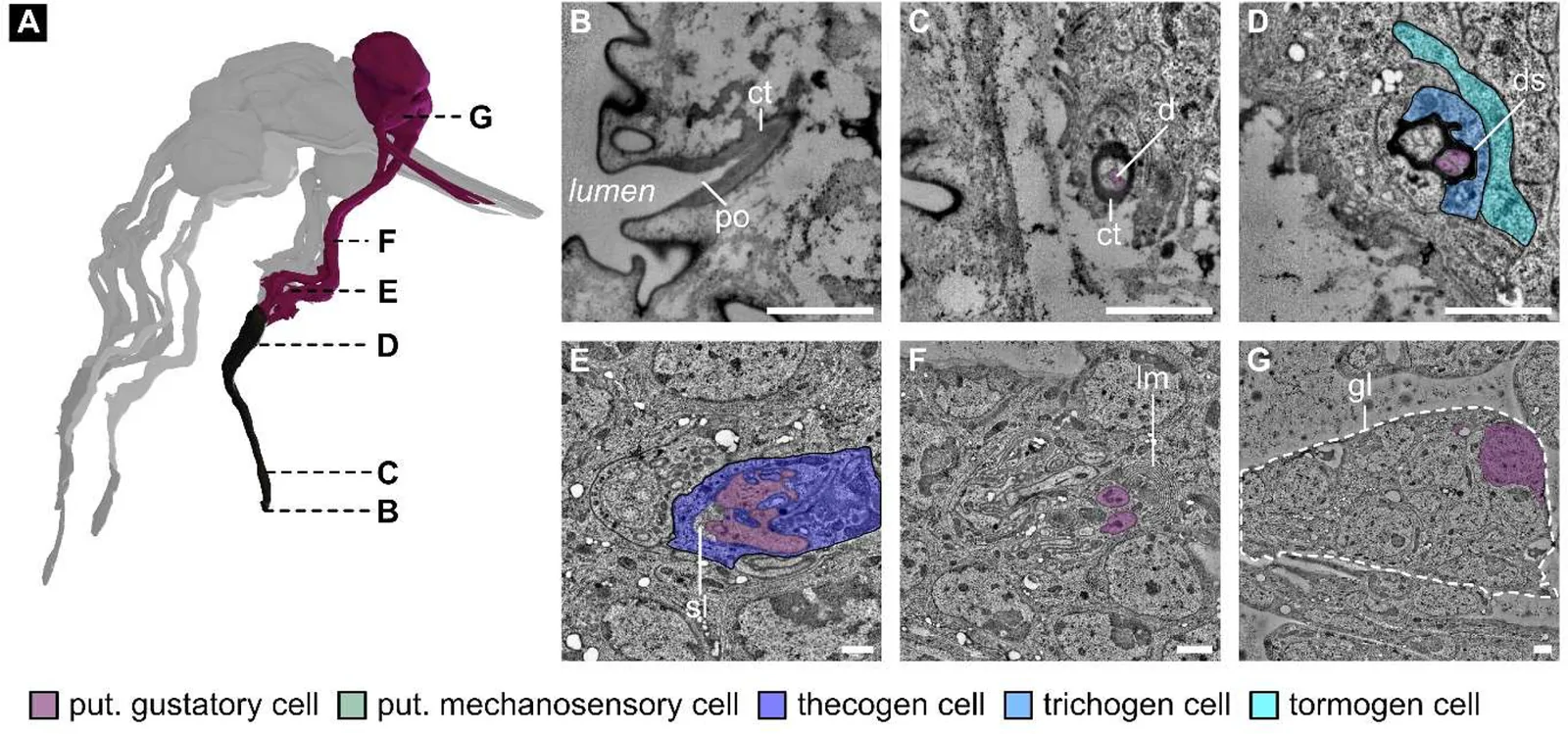
DPS-T_4_. Internal morphology of the pit sensillum 4 (T_4_) of the dorsal pharyngeal sensilla (DPS). (**A**) 3D reconstruction of the T_4_ sensillum. Two sensory neurons (purple) innervate T_4_. The sensillum shares a terminal pore to the pharyngeal lumen with the pit sensillum T_5_ (see Figure 3—figure supplement 7). (**B–G**) Serial scanning transmission electron microscopy (STEM) sections of the T_4_ sensillum. (**B**) The T_4_ sensillum sits in a small pore at the posterior end of the pharyngeal cavity (ph). (**C**) Its dendrites extend into the pore through the cuticle channel, together with the T_5_ dendrites. (**D**) Further proximal, a dendritic sheath appears that separates the dendrites of T_4_ and T_5_ from each other. The trichogen and tormogen support cells are visible. (**E**) The dendrites ciliary structures and the thecogen cell are visible; the dendritic sheath is absent at this level. The dendrites are bathed in the sensillum lymph. (**F**) The sheath cell is highly lamellated at this level. (**G**) Section of the VPS ganglion. One cell body and one dendrite of the T_4_ neurons are visible and lie inside the ganglion. Abbreviations: po – pore; ct – cuticle tube; d – dendrites; ds – dendritic sheath; sl – sensillum lymph; lm – lamellae; gl – ganglion. Scale bars: B–G: 1 µm.

**Figure 3—figure supplement 7.**
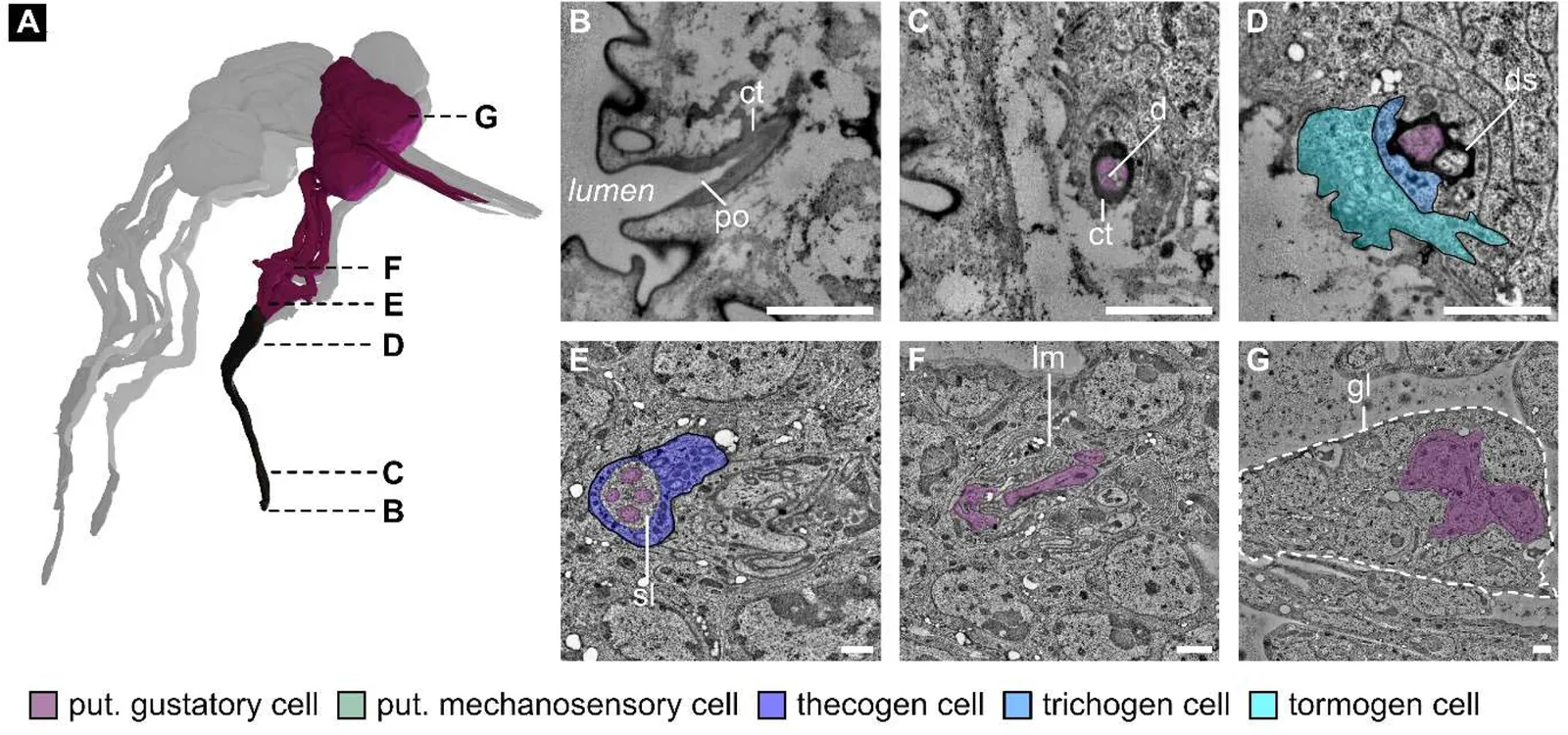
DPS-T_5_. Internal morphology of the pit sensillum 5 (T_5_) of the dorsal pharyngeal sensilla (DPS). (**A**) 3D reconstruction of the T_5_ sensillum. Four sensory neurons (purple) innervate T_5_. The sensillum shares a terminal pore to the pharyngeal lumen with the pit sensillum T_4_ (see Figure 3—figure supplement 6). (**B–G**) Serial scanning transmission electron microscopy (STEM) sections of the T_5_ sensillum. (**B**) The T_5_ sensillum sits in a small pore at the posterior end of the pharyngeal cavity (ph). (**C**) Its dendrites extend into the pore through the cuticle channel, together with the T_4_ dendrites. (**D**) Further proximal, a dendritic sheath appears that separates the dendrites of T_4_ and T_5_ from each other. The trichogen and tormogen support cells are visible. (**E**) The dendrites’ ciliary structures and the thecogen cell are visible; the dendritic sheath is absent at this level. The dendrites are bathed in the sensillum lymph. (**F**) The sheath cell is highly lamellated at this level. (**G**) Section of the VPS ganglion. Three cell bodies and one dendrite of the T_5_ neurons are visible and lie inside the ganglion. Abbreviations: po – pore; ct – cuticle tube; d – dendrites; ds – dendritic sheath; sl – sensillum lymph; lm – lamellae; gl – ganglion. Scale bars: B–G: 1 µm.

Nevertheless, each DPS sensillum possesses its own set of support cells and its own dendritic sheath that separates the dendrites of the sensilla from each other and creates an individual sensillum lymph cavity (Figure 3—figure supplements 3–7D–F), but for T1–T3 and T4+T5, all trichogen cells generate the respective common cuticle tube. This finding is in contrast to the proposed organization of the LSO (Nayak and Singh, 1983), which suggests that the eight sensory cells are accompanied by a shared set of three support cells. These differences may arise from the superior detail and completeness of a whole volume EM stack compared to conventional EM.

The thecogen cells display varying degrees of lamellation, ranging from absent in P/S_1_ and P/S_2_, to weak in T_1_, T_2_, and T_3_, and pronounced in T_4_ and T_5_ (Figure 3—figure supplements 1–7E, F). All pit sensilla neurons are most likely gustatory, as their dendrite endings are in contact with the pharyngeal lumen and do not display another morphological feature, indicating a different sensory modality. The DPS papilla/spot sensilla most likely serve a mechanosensory function, as their dendrite tips contain a tubular body, and the dendritic sheath does not merge into a cuticle tube (Figure 3—figure supplement 1 and 2C, D). Most somata of neurons innervating the DPS lie in the DPS ganglion (DPSG) (Figure 3—figure supplements 2–7G). Only the soma of P/S_1_ lies nearby outside the ganglion (Figure 3—figure supplement 1G). All DPS neurons send their axons to the SEZ via the labral nerve (LrN).

### Dorsal pharyngeal organ

**Figure 4.**
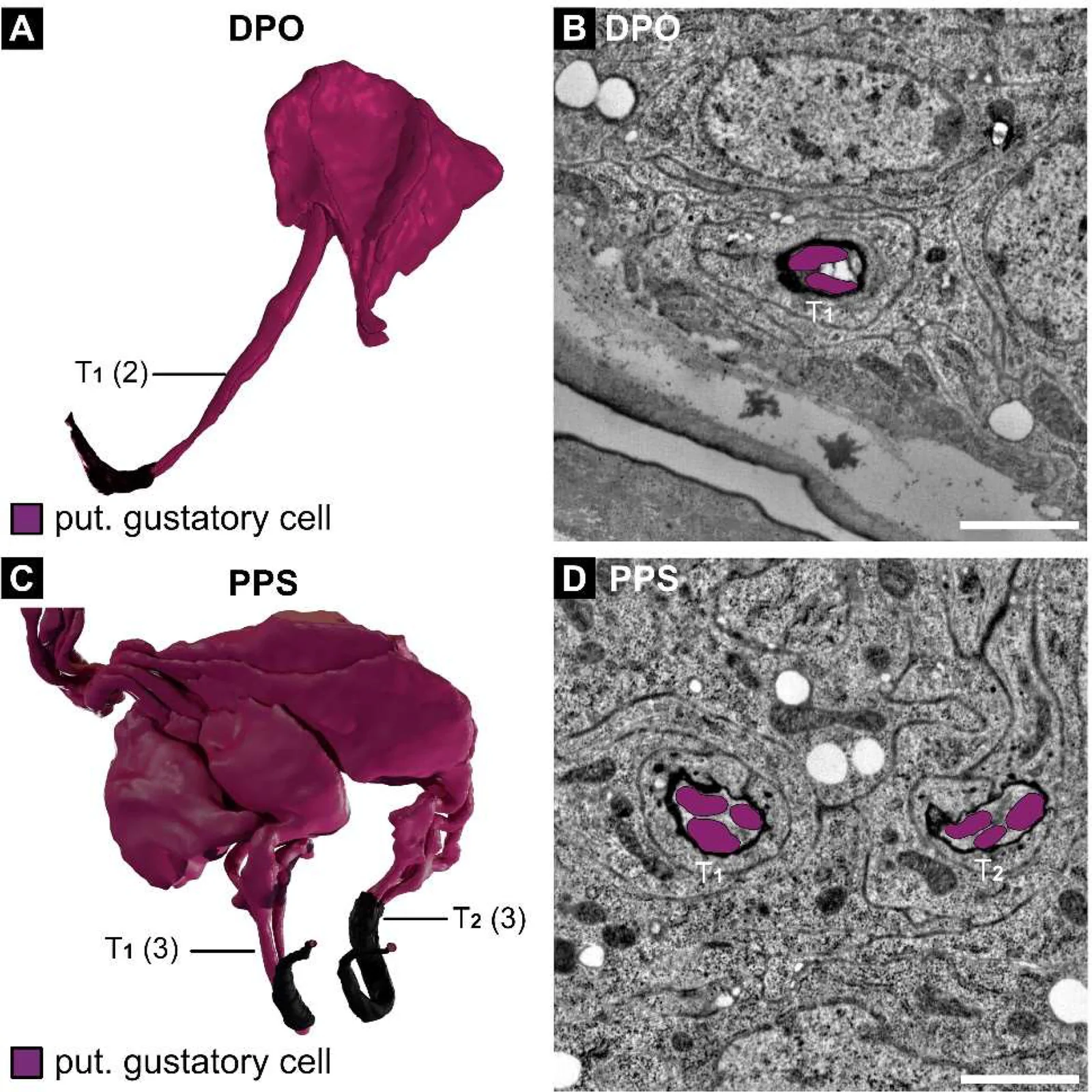
Main cellular configuration of the dorsal pharyngeal organ (DPO) and posterior pharyngeal sensilla (PPS). (**A**) 3D reconstruction of the sensory neurons innervating the DPO. The DPO is a bilaterally organized organ; only the reconstruction of the sensillum on the left is shown. This sensillum is a pit sensillum (T_1_) that is innervated by two sensory neurons. These are connected through a pore with the pharyngeal lumen. (**B**) Scanning transmission electron microscopy (STEM) section of the DPO. The sensory neurons innervating the sensilla are highlighted and color-coded for their putative sensory function (purple – gustatory). (**C**) 3D reconstruction of the sensory neurons innervating the PPS. The PPS is a bilaterally organized organ; only the reconstructions of sensilla on the left are shown. These sensilla are pit sensilla (T_1_ and T_2_), innervated by three sensory neurons each. Their pores to the pharyngeal lumen are located in close proximity to each other. (**D**) STEM section of the PPS. The sensory neurons innervating the sensilla are highlighted and color-coded for their putative sensory function (purple – gustatory). Ultrastructural features of the outer and inner morphology were used to classify the DPO and PPS sensilla. For a detailed description of DPO and PPS sensilla, see Figure 4—figure supplements 1–3. The nomenclature for sensilla was adapted from previous work (Chu-Wang and Axtell, 1972; Rist and Thum, 2017). Abbreviations: d – dorsal; v – ventral; a – anterior; p – posterior; T – pit sensillum. Scale bars: B: 1 µm; D:1 µm.

The DPO is a small, bilaterally organized structure that contains only one pit sensillum (T_1_) on each side, innervated by two Type I neurons (Figure 4A and B; Figure 4—figure supplement 1A). From the ganglion, the dendrites extend toward a location posterior to the DPS. The pit sensillum features a cuticle tube (Figure 4—figure supplement 1B) and a terminal pore (Figure 4—figure supplement 1C) leading to the pharyngeal lumen. Therefore, the two innervating neurons are most likely gustatory. The sensillum includes the standard repertoire of support cells that enclose the sensory neurons and the sensillum lymph (Figure 4—figure supplement 1D and E). The thecogen cell exhibits weak lamellations (Figure 4—figure supplement 1F). All cell bodies of neurons innervating the DPO are located in the DPO ganglion (DPOG).

**Figure 4—figure supplement 1.**
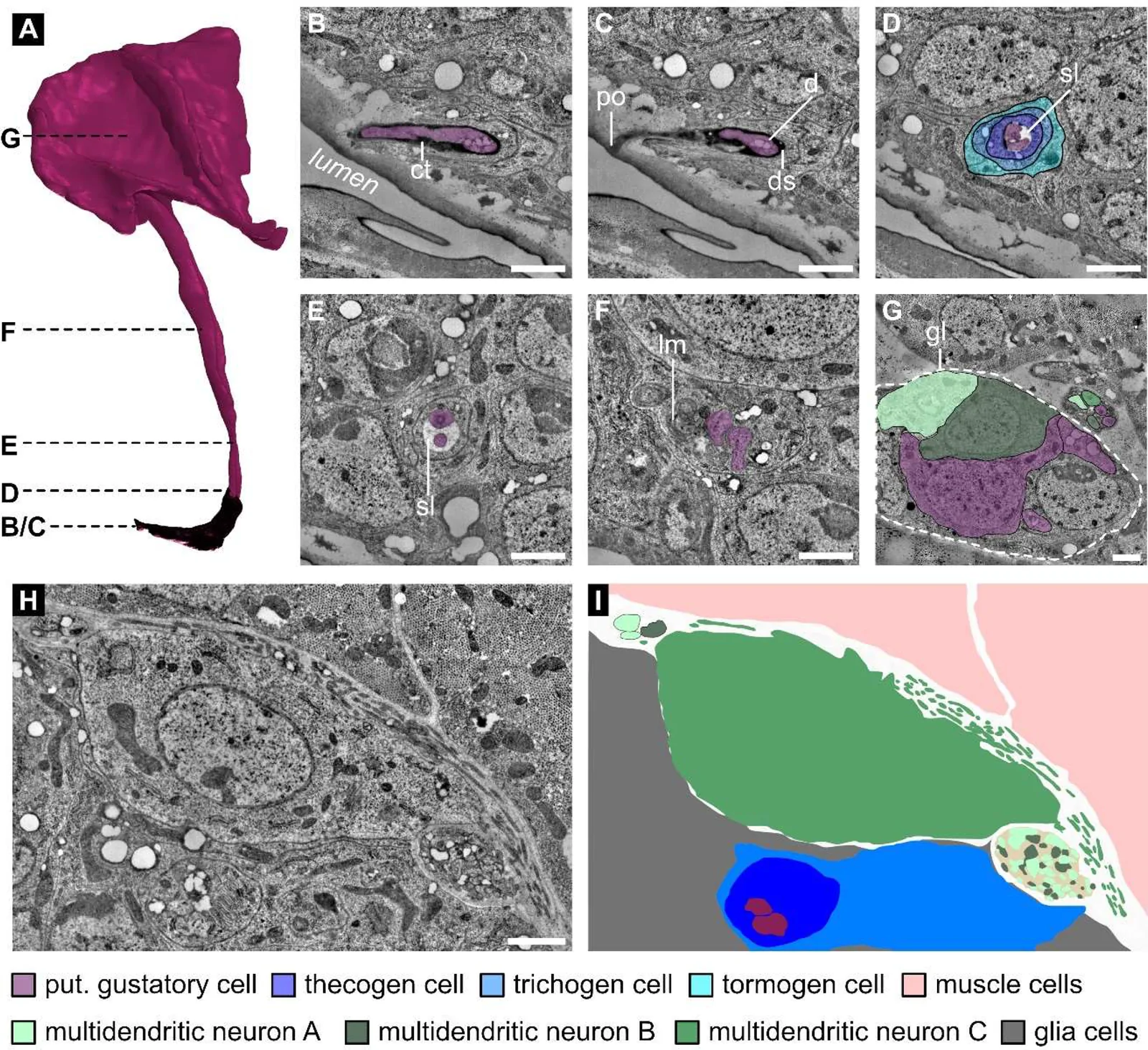
DPO-T1. Internal morphology of the pit sensillum 1 (T_1_) of the dorsal pharyngeal organ (DPO). (**A**) 3D reconstruction of the T_1_ sensillum. Two sensory neurons (purple) innervate T_1_. (**B–G**) Serial scanning transmission electron microscopy (STEM) sections of the T_1_ sensillum. (**B**) The terminal pore is visible. (**C**) The pore is connected to a cuticle channel, which is innervated by the dendrites (**D**). The two ciliary structures (purple) are visible. A dendritic sheath is present but disintegrating at this level. The tips of the dendrites are bathed in the sensillum lymph, enclosed by the thecogen, trichogen, and tormogen support cells. (**E**) The two ciliary structures (purple) are visible. The dendritic sheath is absent at this level. (**F**) The outer dendritic segments pass through the outer support cells. (**G**) Section of the DPO ganglion (DPOG). The cell bodies of the T_1_ neurons and of multidendritic neurons A and B are visible. (H+I) Section of the DPOG; the cell body of multidendritic neuron C is visible, from which multiple dendrites extend in the extracellular space toward the muscle cells. Abbreviations: po – pore; ct – cuticle tube; d – dendrites; ds – dendritic sheath; sl – sensillum lymph; lm – lamellae; gl – ganglion. Scale bars: B–H: 1 µm.

**Figure 4—figure supplement 2.**
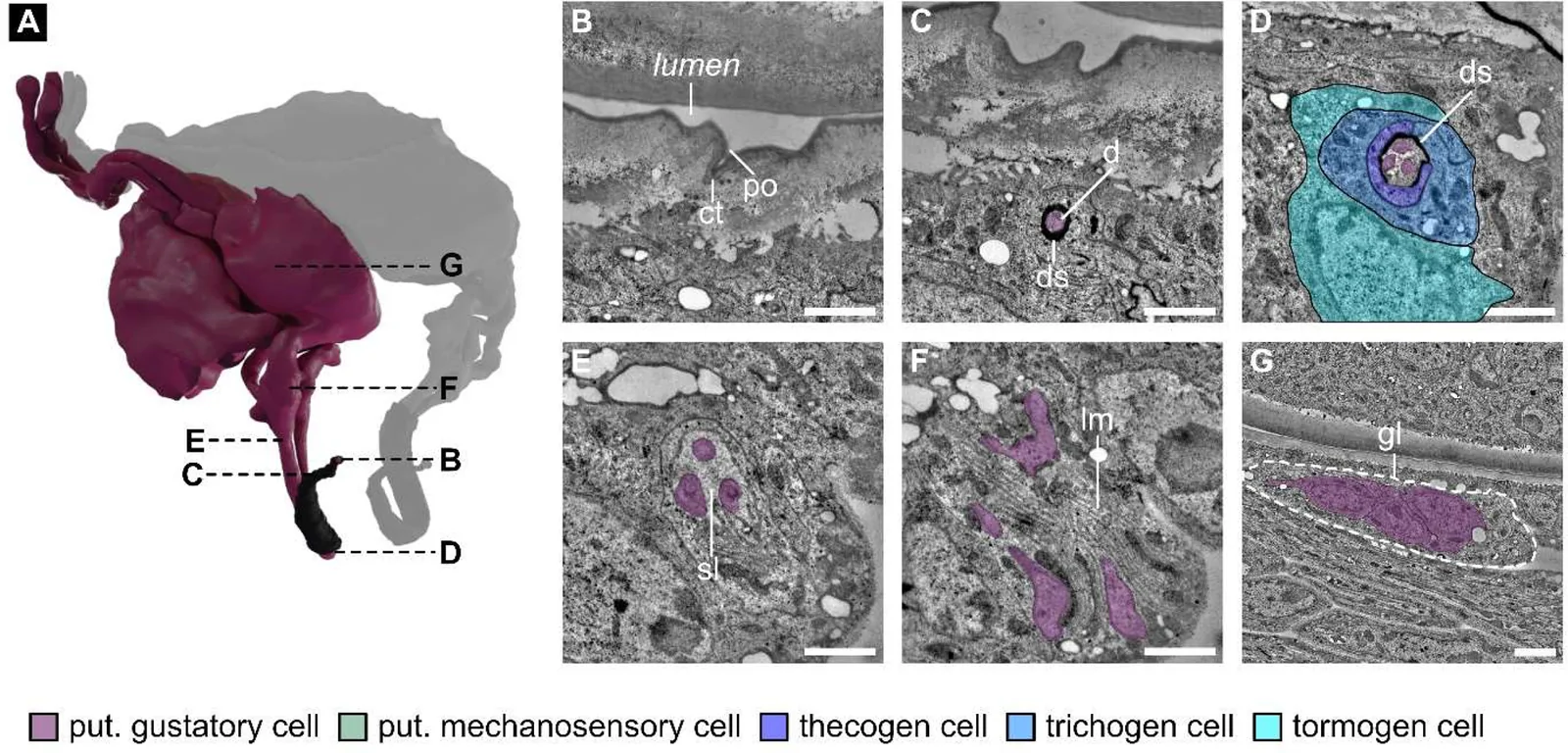
PPS-T_1_. Internal morphology of the pit sensillum 1 (T_1_) of the posterior pharyngeal organ (PPS). (**A**) 3D reconstruction of the T_1_ sensillum. Three sensory neurons (purple) innervate T_1_. (**B–G**) Serial scanning transmission electron microscopy (STEM) sections of the T_1_ sensillum. (**B**) The terminal pore is visible. (**C**) The pore is connected to a cuticle channel, which is innervated by the dendrites (**D**). A dendritic sheath encloses the dendrites but disintegrates at this level. The tips of the dendrites are bathed in the sensillum lymph, enclosed by the thecogen, trichogen, and tormogen support cells. (**E**) The three ciliary structures (purple) are visible. The dendritic sheath is entirely absent at this level. (**F**) The outer dendritic segments pass through the thecogen cell, which is highly lamellated. (**G**) Section of the PPS ganglion (PPSG). The cell bodies of the T_1_ neurons are visible. Abbreviations: po – pore; ct – cuticle tube; d – dendrites; ds – dendritic sheath; sl – sensillum lymph; lm – lamellae; gl – ganglion. Scale bars: B–G: 1 µm.

**Figure 4—figure supplement 3.**
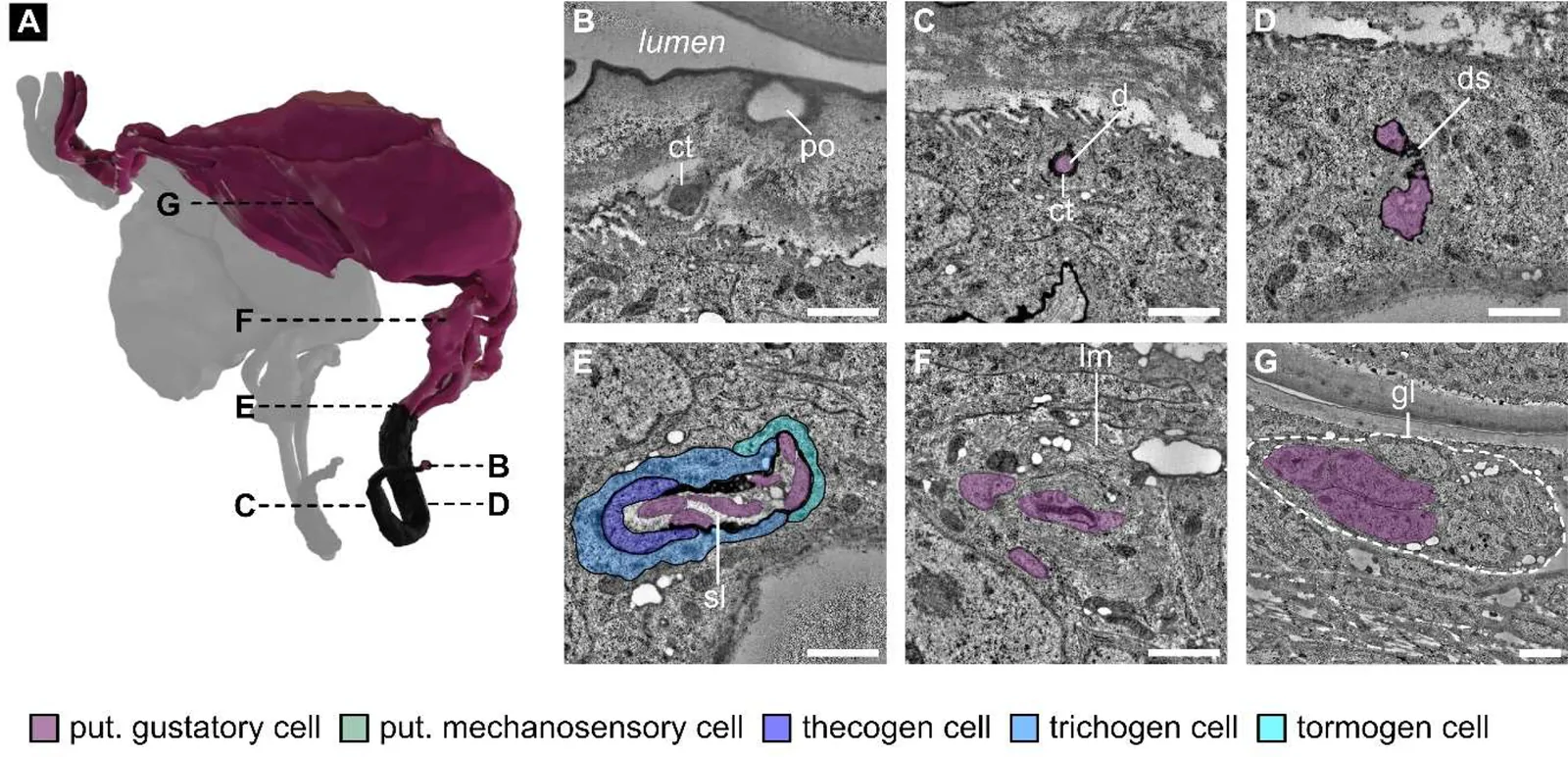
PPS-T_2_. Internal morphology of the pit sensillum 2 (T_2_) of the posterior pharyngeal organ (PPS). (**A**) 3D reconstruction of the T_2_ sensillum. Three sensory neurons (purple) innervate T_2_. (**B–G**) Serial scanning transmission electron microscopy (STEM) sections of the T_2_ sensillum. (**B**) The terminal pore is visible. (**C**) The pore is connected to a cuticle channel, which is innervated by the dendrites (**D**). The dendrites are enclosed by a dendritic sheath (**E**). The three ciliary structures (purple) are visible. A dendritic sheath is present but disintegrating at this level. The tips of the dendrites are bathed in the sensillum lymph and are enclosed by the thecogen, trichogen, and tormogen support cells. (**F**) The outer dendritic segments pass through the thecogen cell, which is highly lamellated. (**G**) Section of the PPS ganglion (PPSG). The cell bodies of the T_2_ neurons are visible. Abbreviations: po – pore; ct – cuticle tube; d – dendrites; ds – dendritic sheath; sl – sensillum lymph; lm – lamellae; gl – ganglion. Scale bars: B–G: 1 µm.

**Figure 4—figure supplement 4.**
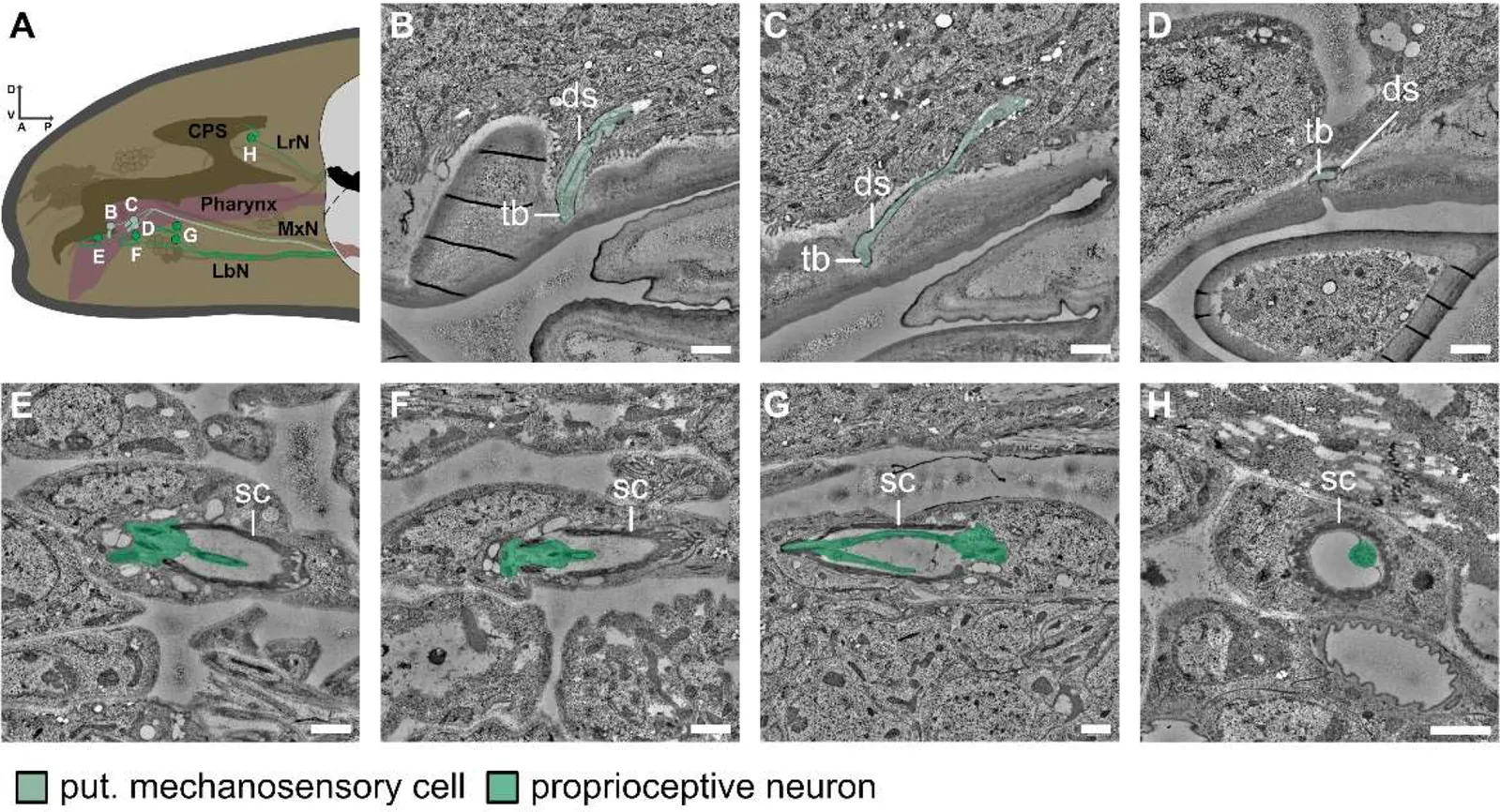
Other internal morphology of the papilla/spot sensilla and chordotonal organs associated with the feeding apparatus. (**A**) Schematic drawing of the larval head region highlighting the position of the additional papilla/spot sensilla and chordotonal organs. (**B–D**) Electron microscopy (EM) images of the dendritic parts of the three papilla/spot sensilla inserting into the pharyngeal lumen. (**E, F**) EM images of the dendritic parts of the four chordotonal organs associated with the feeding apparatus. Sensilla were identified using prominent structures like a dendritic sheath and a tubular body (papilla/spot sensilla) or a sensory neuron inserted into a scolopale (chordotonal organs). Scale bars: B–H: 1 µm. Abbreviations: D – dorsal; V – ventral; A – anterior; P – posterior; CPS – cephalopharyngeal skeleton; LrN – labral nerve; MxN – maxillary nerve; LbN – labial nerve; ds – dendritic sheath; tb – tubular body; sc – scolopale.

Additionally, three multidendritic Type II neurons, here named MD1, MD2, and MD3, are located in the DPOG (Figure 4—figure supplement 1G–I). These neurons extend multiple dendrites into the extracellular space, where, based on their position and morphology, they are likely to perceive internal chemosensory, thermosensory, or mechanosensory signals. The dendrites of MD1 and MD2 wrap around the cell body of MD3 and form a bulbous structure in which multiple dendrites branch and intertwine. This structure resembles mechanoreceptors in human skin, such as Pacinian, Meissner, or Krause corpuscles, and may serve a similar mechanosensory function, although chemosensory or thermosensory roles are also possible (Krause, 1880; Paré et al., 2001; Handler and Ginty, 2021; Ziolkowski et al., 2025). In contrast, the dendrites of MD3 extend toward nearby muscle cells, suggesting a potential role in sensing muscle movements during ingestion, while a chemosensory function cannot be excluded. The markedly different dendritic arborizations of MD1, MD2, and MD3 suggest that these neurons represent distinct sensory types that may be tuned to different modalities, sensitivities, or stimulus directionalities. Although their precise functional roles remain unclear, we propose that these neurons act as internal sensors detecting hemolymph signals and/or mechanical cues associated with muscle activity. All DPOG neurons project their axons to the SEZ via the LrN.

### Posterior pharyngeal sensilla

The PPS is a bilaterally organized structure containing two pit sensilla (T_1_ and T_2_) on each side that are innervated by three Type I neurons each (Figure 4C and D). From the ganglion, the dendrites extend toward the posterior-most location in the pharynx, right before the esophagus (Figure 1B). The pit sensilla display a cuticle tube and a terminal pore to the pharyngeal lumen. Therefore, the three innervating neurons are most likely gustatory. All cell bodies of neurons innervating the PPS lie in the PPS ganglion (PPSG) (Figure 4—figure supplement 2 and 3). All PPSG neurons send their axons to the SEZ via the LrN.

### Additional sensory structures

In addition to the main pharyngeal sense organs, we find three single papilla/spot sensilla associated with the feeding apparatus. These resemble classic mechanosensory sensilla found in the larval segments. They possess a tubular body at their dendritic tip, and a dendritic sheath encloses the dendrite. Their dendrites protrude toward the pharyngeal lumen anterior to the DPS/VPS region, and their axons run with the LbN (Figure 4—figure supplement 4A–D).

Furthermore, four chordotonal organs are also associated with the larval feeding apparatus (Richter et al., 2024). Three lie along the ventral part of the cephalopharyngeal skeleton (CPS), with the anterior two being monodynal (innervated by a single sensory neuron) and the posterior one being heterodynal (innervated by two sensory neurons). Their axons run with the LbN. The fourth one can be found in the dorsal posterior portion of the CPS. It is also monodynal, and its axon runs along the LrN (Figure 4—figure supplement 4A, E–H).

In addition to the Type I sensory neurons organized in the sensilla, we also identified six multidendritic neurons associated with the larval feeding apparatus. As mentioned above, three are located within the VPSG, and three are found in the DPOG.

### Summary/conclusion

The larval pharyngeal sensory system comprises four major organs containing 15 sensilla (four mechanosensory, nine chemosensory, two dual-function), with 35 Type I neurons (6 mechanosensory, 29 chemosensory) and 6 Type II neurons in total. Additional sensory structures include three papilla/spot sensilla and four chordotonal organs, distributed across the cephalopharyngeal region, completing our understanding of the larval sensory system. For the first time, the exact sensilla and cell numbers for the pharyngeal organs and sensilla have been described, and a functional prediction of their sensory modality has been made.

In total, 124 Type I neurons are running with the major head nerves (MxN – 47; LbN – 14; AN – 37; LrN – 26). Based on our and previous analyses, there are 55 gustatory or chemosensory neurons, 40 mechanosensory or proprioceptive neurons, 21 olfactory neurons, 6 thermosensory neurons, and 2 neurons with unidentified function (for details, see Table 2). We conclude that these neurons, in conjunction with the enteric system, constitute the primary sensory apparatus for making feeding decisions. This includes navigating toward desirable conditions, evaluating the chemical and physical quality of food, and incorporating this information with the internal state of the animal.

**Table 2.** Neuronal composition and putative modality of larval head organs and their nerves.

	Sensilla	Olfactory	Gustatory	Chemo (other)	Thermo	Mechano	Other	Total	Ganglion	Nerve
**DO**	7 OS 3 TS 3 P/S	21 6 5	– – –	– – –	– 6 –	– – 3	– – 2	**21** **6** **5**	DOG DOG DOG	AN AN AN
**TOdo**	1 P 1 P_mod_ 1 P/S	– – –	3 – –	– – –	– – –	– 1 1	– – –	**3** **1** **1**	DOG TOG TOG	AN MxN MxN
**TOdi**	5T 2 K 3 P 1 P/S	– – – –	12 – 8 –	– 2 – –	– – – –	2 – 3 1	– – – –	**14** **2** **11** **1**	TOG TOG TOG TOG	MxN MxN MxN MxN
**VO**	1T 3 P/S	– –	2 –	– –	– –	– 3	– –	**2** **3**	VOG VOG	MxN MxN
**LO**	2 P/S	–	–	–	–	2	–	**2**	LOG	MxN
**DPS**	5T 2 P/S	– –	15 –	– –	– –	– 2	– –	**15** **2**	DPSG DPSG	LrN LrN
**DPO** *	1T	–	2	–	–	–	–	**2**	DPOG	LrN
**PPS**	2T	–	6	–	–	–	–	**6**	PPSG	LrN
**VPS** *	2 P 1 P_mod_ 1 H 1T	– – – –	4 – – 2	– – – –	– – – –	2 1 1 –	– – – –	**6** **1** **1** **2**	VPSG VPSG VPSG VPSG	LbN LbN LbN LbN
**P/S – Ext**	1 P/S	**–**	**–**	**–**	**–**	**1**	**–**	**1**	–	AN
**P/S – Ext**	5 P/S	**–**	**–**	**–**	**–**	**5**	**–**	**5**	–	MxN
**P/S – Ph**	3 P/S	**–**	**–**	**–**	**–**	**3**	**–**	**3**	–	MxN
**ChO–DO**	1	**–**	**–**	**–**	**–**	**1**	**–**	**1**	–	AN
**ChO–TO**	1	**–**	**–**	**–**	**–**	**2**	**–**	**2**	–	MxN
**ChO–CPS**	1	**–**	**–**	**–**	**–**	**1**	**–**	**1**	–	LrN
**ChO–Ph**	3	**–**	**–**	**–**	**–**	**4**	**–**	**4**	–	LbN
**TOTAL**	**63**	**21**	**54**	**2**	**6**	**39**	**2**	***124***		
**DOG**	14	21	3	–	6	3	2	**35**		
**TOG**	13	–	20	2	–	8	–	**30**		
**VOG**	4	–	2	–	–	3	–	**5**		
**LOG**	2	–	–	–	–	2	–	**2**		
**DPSG**	7	–	15	–	–	2	–	**17**		
**DPOG**	1	–	2	–	–	–	–	**2**		
**PPSG**	2	–	6	–	–	–	–	**6**		
**VPSG**	5	–	6	–	–	4	–	**10**		
**Single P/S**	**9**	**–**	**–**	**–**	**–**	**9**		**9**		
**ChOs**	**4**	**–**	**–**	**–**	**–**	**5**	**–**	**8**		

Table updated from Python and Stocker, 2002. This summary presents a synopsis of previously reported cell numbers and identities (Singh and Singh, 1984; Campos-Ortega and Hartenstein, 1985; Schmidt-Ott et al., 1994; Rist and Thum, 2017; Richter et al., 2024), as well as the data presented in this work.

Abbreviations: DO – dorsal organ; TO – terminal organ; TOdo – dorsal group of the terminal organ; TOdi – distal group of the terminal organ; VO – ventral organ; LO – labial organ; DPS – dorsal pharyngeal sensilla; DPO – dorsal pharyngeal organ; PPS – posterior pharyngeal sensilla; VPS – ventral pharyngeal sensilla; P/S – papilla/spot sensillum; ChO – chordotonal organ; OS – olfactory sensillum; TS – thermosensory sensillum; P – papillum sensillum; P_mod_ – modified papillum sensillum; T – pit sensillum; K – knob sensillum; H – hair sensillum; DOG – dorsal organ ganglion; TOG – terminal organ ganglion; VOG – ventral organ ganglion; LOG – labial organ ganglion; DPSG – dorsal pharyngeal sensilla ganglion; DPOG – dorsal pharyngeal organ ganglion; PPSG – posterior pharyngeal sensilla ganglion; VPSG – ventral pharyngeal sensilla ganglion; CPS – cephalopharyngeal skeleton; Ph – pharynx; Ext – external; AN – antennal nerve; MxN – maxillary nerve; LrN – labral nerve; LbN – labial nerve.

* In the VPS and DPO ganglia, there are three cell bodies of multidendritic neurons each, which in sum is in accordance with previously reported cell numbers. Multidendritic neurons (MDNs) have generally not been considered here; however, based on previously reported cell numbers in the head nerves (Miroschnikow et al., 2018), we propose that there are approximately 25 sensory MDNs (per side) innervating the head region through the MxN, AN, and PaN. Additionally, there are 36 sensory MDNs (in total) projecting through the larval vagus nerve (VN) and innervating the aorta, esophagus, and gut.

Future work will be needed to trace the peripheral sensory system of the larval pharynx from the sensory organs to the brain via the axons of its associated sensory neurons and to align their central terminals with those identified in previous studies, including Miroschnikow et al., 2018. Such analyses will provide a basis for defining the sensory subcircuits of the larval feeding network and for understanding how their wiring principles transform food-related information into appropriate behavioral outputs.

In conclusion, this comprehensive characterization of the *Drosophila* larval pharyngeal sensory system provides an unprecedented mapping of neuronal architecture and function. This detailed neuroanatomical foundation will help to gain new insights into sensorimotor integration, decision-making circuits, and the neural basis of adaptive behaviors in response to environmental changes.

## Materials and methods

### Whole larval volume

Sensilla and neuron reconstruction were performed on a STEM volume of a whole first instar larva; technical details of its generation are provided in Schoofs et al., 2024 and Peale et al., 2024. Briefly, a first instar larva was sectioned into 4866 sections of 34 nm thickness that were placed on 1622 grids (3 per grid), using a system for automated section cutting and pickup called iTome (short for interferometric microtome). For imaging of the sections, the grids were post-stained with uranyl acetate and lead acetate and then placed into a Zeiss Ultra 55 (modified for operation as a STEM). The micrographs were then reassembled into one dataset (Peale et al., 2024).

We identified sensory structures by scanning the dataset in the pharyngeal region, thereby recognizing dendritic processes toward the pharyngeal lumen. Localization of all sensilla and neurons in the pharyngeal region was done using CATMAID (http://www.catmaid.org; Saalfeld et al., 2009).

### Image processing

We extracted smaller volumes of the sensory organs from the entire larval volume. The obtained image stacks were imported into Amira (Thermo Fisher Scientific, v2019). Since the whole larval volume was already aligned, the stacks were only slightly realigned by manual correction. In Amira, structures of interest were segmented and transformed into 3D objects. Next, the segmentations were imported to Blender (Blender Institute, Amsterdam), where the 3D reconstructions were manually finished using the preliminary segmentation as a template.

## Data Availability

The STEM datasets are publicly available on DRYAD as of the date of publication (https://doi.org/10.5061/dryad.2fqz6135z). Information on the reused whole larval L1 dataset can be found in Schoofs et al., 2024 (https://doi.org/10.1016/j.cub.2024.08.025) and Peale et al., 2024 (https://doi.org/10.1101/2024.07.02.601671). The following dataset was generated: RichterV TriphanT CardonaA ThumAS 2026Data from: Morphology and ultrastructure of pharyngeal sense organs of *Drosophila* larvaeDryad Digital Repository10.5061/dryad.2fqz6135zPMC1369410142912436

## Additional files

Supplementary file 1. Comparison of pharyngeal and cibarial organs in different insect species.

MDAR checklist
