# Supplementary material for: Morphology and ultrastructure of pharyngeal sense organs of Drosophila larvae
Source: eLife. 2026 Oct 23;14:RP108036. doi: 10.7554/eLife.108036 (PMC13694101; doi:10.7554/eLife.108036)
Supplement: Supplementary file 2 [file elife-108036-supp1.docx]

| **Taxon** | **Species** | **organ name** | **sensillum type** | **sensilla** | **neurons** | **sensory modality** | **references** |
| --- | --- | --- | --- | --- | --- | --- | --- |
| ***DIPTERA*** | *D. melanogaster* (larvae) | DPS | papilla/spot sensilla, pit sensilla | 7 | 17 | Mechanosensory; Gustatory | Gendre 2004; this work |
|  |  | DPO | pit sensilla | 1 | 2 (+3*) | Gustatory | Gendre 2004; this work |
|  |  | VPS | pit sensilla, papillum sensilla, hair sensilla | 5 | 10 | Mechanosensory; Gustatory | Gendre 2004; this work |
|  |  | PPS | pit sensilla | 2 | 6 | Gustatory | Gendre 2004; this work |
|  |  | single sensilla | papilla/spot sensilla | 3 | 3 | Mechanosensory | Gendre 2004; this work |
|  | *D. melanogaster* (adult) | LSO | bristle sensilla, hair sensilla, hairless sensilla | 9 | 18 | Mechanosensory; Gustatory | Nayak and Singh 1983; Gendre 2004 |
|  |  | VCSO | bristle sensilla, hair sensilla, hairless sensilla | 3 | 8 | Gustatory | Nayak and Singh 1983; Gendre 2004 |
|  |  | DCSO | bristle sensilla, hair sensilla, hairless sensilla | 2 | 6 | Gustatory | Nayak and Singh 1983; Gendre 2004 |
|  |  | ventral and dorsal row sensilla | fishtrap bristle sensilla | 18-23 | 1 | Mechanosensory | Nayak and Singh 1983; Gendre 2004 |
|  | *C. vicinia (*larvae) | LbO (=LSO) | N/A | N/A | N/A | Mechanosensory | Ludwig 1949; Hückesfeld 2010 |
|  | *C. vicinia* (adult) | CO | basiconic, trichoid and campaniform sensilla | 24 | N/A | Mechanosensory; Gustatory | Rice 1973 |
|  | *M. domestica* (larva) | LSO | N/A | N/A | N/A | Mechanosensory; Gustatory | Ranade, 1967 |
|  | *A. aegypti (*adult) | Cibarial Sensilla | Papilla sensilla, campaniform sensilla | 9 (female), 7 (male) | N/A | Mechanosensory; Gustatory | McIver 1981; Lee 1983; Baik 2020 |
| ***LEPIDOPTERA*** | *M. sexta* (larvae) | Epipharyngeal sensilla | N/A | 1 | 3 | Gustatory | Glendinning 2000 |
|  | *Heterogynis penella* (larvae) | Cibarial sensilla | N/A | N/A | N/A | N/A | Vegliante 2005 |
| ***COLEOPTERA*** | *L. decemlineata* (larvae) | Epipharyngeal sensilla | N/A | 10 | N/A | Gustatory | Messchendorp 1998 |
| ***HEMIPTERA*** | *R. proxilus* (adult) | Pharyngeal Organ (PO) | Short-peg sensilla | 8** | N/A | Gustatory | Ortega-Insaurralde 2024 |
| ***HYMENOPTERA*** | *A. mellifera* (adult) | Epipharyngeal sensilla | N/A | ~180 | N/A | Gustatory | Galic 1971 |
| ***HYMENOPTERA*** | *A. mellifera* (adult) | Hypopharyngeal sensilla | N/A | ~90 | N/A | Gustatory | Galic 1971 |
| ***ORTHOPTERA*** | *Acheta domesticus* (adult) | Pharyngeal neurons | N/A | N/A | N/A | N/A | Kirby 1984 |
| ***ODONATA*** | *Ischnura elegans (adult)* | Epipharyngeal sensilla | hair sensilla, peg sensilla | 30** (hair); 50** (pegs) | N/A | Mechanosensory; Gustatory | Rebora 2014 |
| **** multidendritic neurons; ** unpaired*** | | | | | | | |
