# Supplementary material for: Morphology and ultrastructure of pharyngeal sense organs of Drosophila larvae
Source: eLife. 2026 Oct 23;14:RP108036. doi: 10.7554/eLife.108036 (PMC13694101; doi:10.7554/eLife.108036)
Supplement: Peer review history

# Editor report: eLife Assessment

**DOI:** 10.7554/eLife.108036.3.sa0

**Authors:** Nicolás Pírez

**Affiliations:** Universidad de Buenos Aires - CONICET Argentina

**Keywords:** Exceptional, Fundamental

In this **fundamental** manuscript, Richter et al. present a thorough anatomical characterization of the *Drosophila melanogaster* larval pharyngeal sensory system, which is involved in taste-guided behaviors. This study fills a major gap in the larval sensory map, providing a detailed neuroanatomical foundation for future investigations into sensory circuits and behavior. The **exceptional** data will significantly enrich the field of *Drosophila* neurobiology.

# Referee report: Reviewer #2 (Public review)

**DOI:** 10.7554/eLife.108036.3.sa1

Summary:

The authors wanted to achieve a detailed ultrastructural reconstruction of the gustatory sensory organs in the Drosophila pharynx. Using serial EM and the associated bioinformatics tools they have achieved their goal.

Strengths:

Given the dataset, finding presented are solid and will be an important work of reference for the future.

Comments on revised version.

The authors have well responded to my previous comments and added text and figure material.

# Author comment: Author response

**DOI:** 10.7554/eLife.108036.3.sa2

**Authors:** Vincent Richter, Tilman Triphan, Albert Cardona, Andreas S Thum

**Affiliations:** Leipzig University Leipzig Germany; Leipzig University Leipzig Germany; University of Cambridge Cambridge United Kingdom; Leipzig University Saxony Germany

The following is the authors’ response to the original reviews.

**Public Reviews:**

**Reviewer #1 (Public review):**

Summary:

The authors provide a detailed ultrastructural analysis of the larval pharyngeal sensory organs, including the dorsal pharyngeal sensilla, dorsal pharyngeal organ, ventral pharyngeal sensilla, and posterior pharyngeal sensilla. Using electron microscopy and 3D reconstruction, Richter et al., present a comprehensive mapping and classification of pharyngeal sensory structures, defining the morphological type of pharyngeal sensilla based on ultrastructure and generating a neuron-to-sensillum map. These findings significantly advance our understanding of internal larval sensory systems and establish a robust framework for future functional studies in coordination with external sensory systems.

Strengths:

The application of high-resolution electron microscopy and 3D imaging analysis successfully overcomes technical challenges associated with visualizing deep internal structures. This enables an unprecedented level of anatomical detail of the larval pharyngeal sensory system. Thus, the study complements and completes existing maps of larval sensory circuits, contributing a comprehensive neuroanatomical characterization of larval sensory input pathways. These insights will inform future studies on larval behavior, sensory processing, and may also have applied relevance for insect control strategies.

Weaknesses:

While the manuscript is concise, clearly written, and methodologically rigorous, it primarily addresses a specialized readership with expertise in insect neuroanatomy.

We thank the reviewer for the positive assessment of our study and for the helpful suggestions. In response, we have clarified the visual presentation of the pharyngeal sense organs in Figure 1, expanded the discussion of adult pharyngeal sensory systems, briefly broadened the comparison to other insect species, checked and corrected the scale bars, and added further methodological detail where appropriate.

**Reviewer #2 (Public review):**

Summary:

This manuscript documents the structure of the pharyngeal nervous system of the Drosophila larva. The authors wanted to achieve a detailed ultrastructural reconstruction of the gustatory sensory organs in the Drosophila pharynx. Using serial EM and the associated bioinformatics tools, they have achieved their goal. The paper is written clearly and illustrated beautifully with 3D models and annotated sections. The data will significantly enrich the field of Drosophila neurobiology.

Strengths:

Given the dataset, the findings presented are solid and will be an important work of reference for the future.

Weaknesses:

Previous work, including EM, on the pharyngeal sensory organ is not sufficiently referenced and used for comparison with the data presented in this study.

We are grateful for the reviewer’s thoughtful comments and for the suggestion to strengthen the historical and comparative context of the work. We have revised the introduction to better acknowledge and discuss the relevant previous EM-based literature on adult and larval internal gustatory sensilla, clarified the organization of the shared pore structure in T1–T3, highlighted the DPO multidendritic neurons more explicitly, and added a comparison that emphasizes the added value of the complete serial EM dataset.

**Recommendations for the authors:**

**Reviewer #1 (Recommendations for the authors):**

(1) For improved clarity, highlight the pharyngeal sense organs in Figure 1B. Consider using the color schemes to differentiate between peripheral and internal sensory organs.

We thank the reviewer for this helpful suggestion. We have revised Figure 1 to more clearly separate the pharyngeal sense organs from the external sense organs in the head region. This revision improves visual clarity and accessibility for readers.

(2) In reference to lines 80-84, expand the discussion to address how future studies could explore the conserved morphological and functional characterization of the adult pharyngeal sensory system.

We appreciate this suggestion and have expanded the discussion accordingly. We now briefly address how future work could compare the larval and adult pharyngeal sensory systems to examine conserved morphological and functional features.

(3) To broaden the manuscript's appeal and emphasize its relevance beyond Drosophila, briefly discuss similarities, differences, or conserved roles of pharyngeal sensory systems in other insect species.

Thank you for this valuable recommendation. We have added a paragraph placing the Drosophila pharyngeal sensory system in a broader insect context, including similarities, differences and potential conservation across species.

(4) Recheck the scale bars in all figures, including the supplemental material.

We thank the reviewer for pointing this out. We carefully rechecked all scale bars across the main and supplemental figures and corrected the missing ones.

(5) Consider including additional details on image processing or provide appropriate citations for further reading.

We appreciate this suggestion. We have expanded the methods section to include additional information on technical details and provide the relevant reference for further reading.

**Reviewer #2 (Recommendations for the authors):**

(1) Line 57ff: The previous literature describes internal gustatory sensilla in considerable detail.

(a) Adult: These sensilla form three complexes, the labral sensory organ, and the ventral and dorsal cibarial sensory organ (Nayak & Singh, 1983, 1985; Singh, 1997; Stocker & Schorderet, 1981; Kendroud et al., 2017). The work by Nayak and Sing includes TEM and presents detailed EM-based schematics. This should be referenced and discussed.

(b) Larva: Gendre et al. 2004, describes the internal gustatory organs and relates them to their adult counterparts:

- Dorsal pharyngeal sense organ (DPS) and dorsal pharyngeal organ DPO are the forerunners of adult labral and ventral cibarial sensory organs

- Posterior pharyngeal sensory organ (PPS) is the forerunner of the adult dorsal cibarial sensory organ

- Ventral pharyngeal sensory organ (VPS), derived from the labial segment, undergoes apoptosis during metamorphosis

This work, connecting larva and adult (and containing detailed diagrams comparing adult and larval pharyngeal sensilla) should be presented in the introduction.

We thank the reviewer for this important comment. We have revised the introduction to better cite and discuss previous EM-based studies of internal gustatory sensilla in both adult and larval stages, and we now place our findings more explicitly in the context of this prior work.

(2) Line 180: the relationship between the ending of T1-T3 in one shared pore, and the individually wrapped sensilla should be explained; maybe a simple diagram would help. I did not understand how it works. Normally, in a gustatory sensillum, you have one or more sensory neurons, surrounded by thecogen, trichogen, and tormogen cells. The trichogen generates the shaft with the pore at its tip. Now here, in T1-T3, you have three sets of thecogen/trichogen/tormogen. Do all three trichogen cells somehow participate in the shaft with the common pore? Or only a single one, and the other two generate no shaft? It is possible this cannot be resolved, but the authors should address the problem and suggest a possible scenario.

We appreciate the reviewer’s concern and agree that this point required clarification. We have revised the relevant text to better explain the organization of T1-T3 and their shared pore and the organization of the support cells.

(3) Line 205: the DPO multidendritic neurons with dendrites into the hemolymph should be shown; in Figure S4G, I could see only cell bodies. These MD neurons in the gustatory system are, I believe, a true novelty and should be emphasized more if the material allows (text figure!)

Thank you for highlighting this point. We have revised the results and supplementary material to show these neurons more clearly and to emphasize their novelty and potential relevance to the pharyngeal sensory system.

(4) A somewhat detailed comparison between the ultrastructure of the DPS as extracted from the serial EM stack of this study, and the conclusions of Nayak and Singh 1983 as depicted in their diagram Figure 7a would be productive. The idea being: what additional details can (only) a complete EM stack provide, compared to conventional EM.

We appreciate this suggestion. Rather than directly comparing larval and adult structures in detail, we now emphasize what the complete serial EM dataset adds beyond conventional single-section EM, namely a more comprehensive and complete reconstruction of the sensory organs and associated cell types (multidendritic neurons, papilla sensilla, and chordotonal organs that were not described before, organization of support cells)

(5) To round off the work and connect it to the previously published analysis of gustatory terminal arborizations and connectivity in the brain (Miroschnikow et al.,2018), it would be helpful to add an analysis of the distribution of axons from the different sensilla in the nerves. Miroschnikow analyzes the central terminations of the same sense for which the peripheral structure is described here, only that in their L1 connectome, the periphery was cut off. Do the findings of the current study match their predictions, as to the number of sensory neurons, etc? It should be possible to follow, even at the lower resolution of the dataset presented here, to follow axons of sensory neurons through the nerves to the neuropil entry, and thereby make the connection. I consider this to be of great importance for the field, for authors who want to use the data of this study, and the Miroschnikow et al analysis, for their own studies.

We thank the reviewer for this thoughtful and constructive suggestion. We fully agree that linking the peripheral sensory anatomy described in this study to the central projections analyzed by Miroschnikow et al. would be highly valuable and of broad interest. However, a systematic analysis of axon distributions from the different sensilla through the nerves to their neuropil entry points is beyond the scope of the present work. Owing especially to the dataset’s resolution and inherent limitations, tracing the connections from sensory organs through the nerves to their projections in the brain is technically highly challenging and extremely time-consuming, since much of the process would need to be performed manually. We therefore do not include a detailed comparison with the predictions from Miroschnikow et al. in this manuscript. Nevertheless, we appreciate that such an analysis would be an important next step for the field and a useful resource for future studies.

We are grateful for the reviewers’ thoughtful feedback, which has helped us improve the manuscript substantially. We hope that the revised version addresses the concerns raised and better conveys the significance of our work.
